# Bidirectional Relationship Between Mental Health and Sports Injury in Adolescents: A Systematic Review and Meta-analysis

**DOI:** 10.1007/s40279-025-02379-z

**Published:** 2026-01-13

**Authors:** Athena R. W. Chow, Mirela Zaneva, Layla Rashid, Catherine Wheatley, Constantin Coussios, Robert Hepach, Lucy Bowes

**Affiliations:** 1 Department of Experimental Psychology, University of Oxford, Life and Mind Building, South Parks Road, Oxford, OX1 3EL UK; 2 Christ Church College, University of Oxford, Oxford, UK; 3 Nuffield Department of Clinical Neurosciences, University of Oxford, Oxford, UK; 4 Institute of Biomedical Engineering, Department of Engineering Science, University of Oxford, Oxford, UK; 5 Podium Analytics, London, UK

## Abstract

**Background:**

Sports injuries are linked to negative impacts on mental health and well-being, including depression, anxiety, stress and lower quality of life. Conversely, poor mental health and well-being have been found to increase the risk of sports injury, injury severity and time taken to recover. Although existing research indicates these associations in athletes broadly, the nature and directionality of these relationships among adolescents are not well characterised. A related limitation is that much of the existing evidence, in both children and adult athletes, is cross-sectional in design, limiting our understanding of causal directionality. Given the high rates of sports participation and the specific risk factors for injury in this demographic, as well as the growing concern about adolescents’ mental health and well-being worldwide, this complex relationship warrants greater attention.

**Objective:**

We aimed to examine the bidirectional relationship between sports injuries and mental health and well-being in adolescents aged 10–24 years, and potential mechanisms of this relationship.

**Methods:**

This systematic review and meta-analysis was registered on PROSPERO (ID: CRD42023374807). Literature searches were performed according to PRISMA (Preferred Reporting Items for Systematic reviews and Meta-Analyses) guidelines by searching PsycINFO, Web of Science, ERIC, CINAHL, MEDLINE, Embase, Cochrane Library, SPORTDiscus, PEDro, Elicit and Google Scholar. Articles were included if they were quantitative, published in English between 1990 and 2023, focused on young athletes aged 10–24 years, measured mental health/well-being and were an all-complaints sports injury that ranged across all levels of sport.

**Results:**

Of 397 studies screened, 84 studies were included. The final sample included 221,095 adolescents and young people. A narrative synthesis indicated that sports injuries were associated with worse mental health and well-being in the majority of studies, and vice versa. Meta-analyses revealed that sports injury incidence was significantly associated with worse mental health/well-being (*Z*_*r*_ = 0.34, 95% confidence interval 0.17, 0.50), and concussion incidence was significantly associated with worse mental health/well-being (*Z*_*r*_ = 0.19, 95% confidence interval 0.08, 0.30), with no evidence of publication bias. Conversely, worse mental health/well-being was associated with a significantly increased risk of sports injury incidence (odds ratio = 1.54, 95% confidence interval 1.13, 2.10). However, after accounting for potential publication bias, the pooled association between mental health/well-being and sports injury risk was no longer statistically significant, highlighting the need for caution in interpreting this relationship. Regarding mechanisms, a small number of studies revealed psychosocial factors (e.g. athletic identity, social support) that could influence this reciprocal relationship.

**Conclusions:**

We found evidence for a bidirectional relationship between sports injuries and mental health in adolescent athletes. Further research is needed to elucidate the mechanisms underlying this relationship. Early interventions focusing on supporting mental health after sports injury, and addressing pre-existing mental health issues to reduce the risk of subsequent injury, should be tailored towards psychosocial mechanisms that particularly impact adolescent athletes.

**Supplementary Information:**

The online version contains supplementary material available at 10.1007/s40279-025-02379-z.

## Key Points

**Table:** 

Adolescent athletes who experience sports injuries are more likely to experience poor mental health and well-being; conversely, a growing body of literature has shown that poor mental health and well-being increase the risk of sports injury incidence, severity and time taken to recover.
Our study is the first to provide evidence for a bidirectional relationship between sports injuries and mental health in adolescents, which has not been robustly established or precisely quantified before.
Mental health support and education, particularly longitudinal mental health surveillance monitoring, should be holistically incorporated into sports medicine practices to improve the physical and psychological health of young athletes.

## Introduction

Millions of adolescents participate in organised sports worldwide. Sports participation is generally considered beneficial for both physical and mental health, as well as an important protective factor for adolescent well-being. In addition to fostering physical health, regular participation in sports and physical activity contributes to improved mood, a reduced risk of depression and anxiety, and greater self-esteem among adolescents. These links have become particularly salient since the onset of the coronavirus disease 2019 pandemic, where loss of access to sports and activity opportunities was associated with diminished mental well-being and greater psychological distress for young people because of social isolation, disrupted routines and reduced physical activity [1–5].

Nevertheless, sporting activities are not without risk, and adolescents have been found to experience the highest incidence of traumatic sports-related injuries compared with other age groups (approximately 1.94 vs 0.72 per 1000 exposures in adolescent and adult athletes, respectively) [6]. Compared with adults, adolescents who take part in sport have a heightened risk of injury, with contributing factors including developing cartilage and neuromuscular coordination [7], as well as increased impulsivity and openness to risk taking [8]. There is some evidence that coaching trends towards higher training loads and earlier specialisation may elevate overuse injury risk in young people [9], though the overall contribution of specialisation versus other age-related factors remains unclear [7–9]. Research has traditionally prioritised surveying adult athletes, as well as physical markers of injury recovery, and so the magnitude of the impact of sports injuries on the mental health of young athletes is not well understood [10]. Adolescence is a critical developmental period when mental health problems begin to emerge [11]. Sports injuries in adolescent athletes are linked to psychosocial challenges such as stress, frustration and lower quality of life (QOL), with severe injuries being linked to long-lasting impacts such as loss of self-identity, fear of reinjury and risk of subsequent injury [11, 12]. Recent evidence indicates that paediatric concussion is associated with an increased risk of mental health difficulties, including depression, anxiety and post-traumatic stress, particularly in those with pre-existing mental health concerns. While most affected youth experience an exacerbation of prior mental health issues, only a minority develop new-onset psychiatric diagnoses following concussion [13].

Conversely, emerging evidence from individual studies suggests that mental health disorders in athletes have been found to increase the risk of injury and time taken to recover [14]. However, this has not been robustly established or precisely quantified, particularly in young athletes [10, 14]. Understanding the strength, direction and underpinning mechanisms of this relationship could inform the development of early interventions to reduce the risk of sports injury, mitigate against post-injury drop-out, and promote better sports and mental health outcomes for young athletes overall. There is a pressing need for holistic research that covers the breadth of this field—considering not only physical injuries from a broad “all complaints” position, but also the psychological states associated with them. By integrating these perspectives, we can better address the complexities of young athlete health and develop comprehensive strategies that support both physical health and mental well-being.

To this end, this systematic review and meta-analysis examined the bidirectional relationship between sports injuries and mental health and well-being in adolescents aged 10–24 years worldwide. Taking an all-complaints position on sports injuries, we aimed to answer the following pre-registered research questions: (1) What is the potential impact of mental health and well-being on sports injuries among adolescents and young people? (2) What is the potential impact of sports injuries on mental health and well-being? (3) What are the potential mechanisms of the relationship between sports injuries and mental health and well-being? We included a wide range of mental health and well-being measures that ranged from clinical diagnoses to symptomatology. Finally, we reviewed the evidence of mechanisms that could influence this complex reciprocal relationship.

## Methods

We developed the protocol for this systematic review in adherence with PRISMA-P (Preferred Reporting Items for Systematic review and Meta-Analysis Protocols [15] (Electronic Supplementary Material [ESM]) and preregistered it at PROSPERO (ID: CRD42023374807).

### Search Strategy and Databases

We implemented our search strategy in PsycINFO, Web of Science, ERIC, CINAHL, MEDLINE, Embase, Cochrane Library, SPORTDiscus and PEDro, searching for peer-reviewed work, book chapters and grey literature (e.g. dissertations) published from 1990 to January 2023. We did not search for earlier literature given the use of non-standardised mental health instruments before 1990 [16, 17]. In November 2023, we updated our search using the artificial intelligence-powered academic search engines Elicit and Google Scholar. We provide our search strategy including the full list of search terms in the ESM.

### Eligibility Criteria

We required studies to be published since 1990, in English, with results presented in quantitative terms. We accepted various quantitative designs, including cross-sectional studies, prospective and retrospective cohort studies, quasi-experimental trials and randomised controlled trials (RCTs). While we included cross-sectional studies to maximise relevant data capture, we recognise that only longitudinal and intervention designs can inform directionality in the mental health–injury relationship. Intervention studies, including RCTs, were not included in the meta-analysis, as pre-post intervention data could not be used to directly test the association between mental health/well-being and injury incidence. Instead, these studies were synthesised narratively to provide a complementary context regarding how injuries may influence mental health and well-being outcomes.

We focused on young people aged 10–24 years who play sports. While the World Health Organization defines adolescence as ages 10–19 years, the American Academy of Pediatrics uses 11–21 years as a definition, and other major organisations and recent research increasingly recognise a broader developmental period extending into the early 20s (e.g. ‘young people’ 10–24 years of age). Our selection was substantiated on both biological (earlier puberty onset, extended neurodevelopment) and social (delayed role transitions) grounds, aligning with recent global health literature advocating an expanded definition of adolescence [18]. If a study’s population fell outside this range, we attempted to include a relevant sub-sample corresponding to the inclusion age range, or, if not possible, we included studies where the mean age still fell within 10–24 years.

We included a variety of mental health and well-being measures, as we recognise that mental health encompasses not only the presence or absence of clinical symptoms but also broader aspects of emotional and psychological states. Further, we acknowledge different research traditions may treat terms such as “mental health” and “mental well-being” as describing the same concepts; thus, we aim to be more inclusive in our search strategy to capture any relevant measures. Specifically, we included measures such as clinical diagnoses (e.g. depression) or symptomatology (e.g. self- or clinician-reported internalising problems). Well-being outcomes were assessed by subjective self-report scales, such as life satisfaction and happiness questionnaires.

We defined sports in line with the UN Inter-Agency Task Force (2003) definition, namely that sport includes “all forms of physical activity that contribute to physical fitness, mental well-being and social interaction, such as play, recreation, organised or competitive sport, and indigenous sports and games” [19]. We rely on the World Health Organization’s definition of physical activity: “any bodily movement produced by skeletal muscles that requires energy expenditure … e.g., upper body led activities, inclusive and/or wheelchair-specific sport and activities” [20].

We did not apply any exclusion criteria regarding sports injuries. We accepted both clinician-assessed and self-reported measures of different injuries during sport (both training and competition) as well as in the broader context of sport (during warm-up and breaks), regardless of the specific type of measurement (e.g. time loss, duration or severity of injury) [21]. We opted for broader inclusion criteria regarding both sports injuries and mental health, so as to maximise relevant data capture in an understudied population (young athletes). Later, we examined the relationships of interest based on theoretically comparable grouping (e.g. concussions).

### Study Selection and Data Extraction

Two authors (MZ and AC) used Rayyan to remove duplicates and independently screen abstracts. For both abstract and full-text screening, studies that did not fit inclusion criteria were excluded and disagreements were collaboratively discussed. Where required, arbitration was provided by a third author (LB). We maintained high inter-rater agreement at both the abstract screening stage (*κ* = 0.83, *p* < 0.001) and the full-text screening stage (*κ* = 0.92, *p* < 0.001). LR independently extracted data from all included papers based on a predetermined data extraction form on the studies’ population characteristics, measures of sports injuries, mental health and/or well-being, experimental design and results. AC and MZ independently extracted two separate random samples comprising 10% of the included studies. AC and MZ then independently double-checked all remaining extracted information. Any discrepancies were discussed first amongst AC, MZ and LR, with further arbitration by LB, where required.

### Risk of Bias Including Publication Bias

Each study was independently assessed by two authors (LR, MZ or AC) for risk of bias. We used National Heart, Lung, and Blood Institute’s Quality Assessment Tools for Observational Cohort and Cross-Sectional Studies, Cochrane’s Risk of Bias Tool for randomised trials and the Risk Of Bias In Non-randomized Studies—of Interventions for non-randomised interventional trials. LB arbitrated when required. We further examined whether studies were preregistered. We assessed publication bias with funnel plots and Egger’s test. Funnel plots are scatterplots of study effect sizes against their precision (often the standard error), visually inspecting for symmetry to detect possible publication bias: unbiased meta-analysis will display a symmetrical inverted funnel shape. Egger’s test is a formal regression-based statistical test for funnel plot asymmetry: it regresses the standardised effect sizes on their precision, with a significant intercept indicating asymmetry. Together, these methods help identify whether smaller studies report systematically different effect sizes, which may suggest publication bias.

### Equity, Diversity and Inclusion

We included all relevant studies irrespective of participants’ sex, race/ethnicity, country and socioeconomic level. Our author team comprised 71% female, junior and senior level researchers from multiple disciplines, and individuals from marginalised backgrounds.

### Narrative Syntheses and Meta-analyses

We conducted two narrative syntheses and three meta-analyses following our preregistered research questions to understand: (1) the impact of mental health and well-being on sports injuries and (2) the impact of sports injuries on mental health and well-being. A narrative analysis involves summarising and synthesising the findings of included studies in a descriptive textual format to provide an overview of the evidence when a meta-analysis is not appropriate. Initially, all injuries were grouped together in the analysis; subsequently, concussion was examined separately from other musculoskeletal injuries, as concussion is characterised by a biomechanically induced neural dysfunction affecting the brain distinctly compared to musculoskeletal injuries [22]. The first meta-analysis investigated whether prior mental health and well-being predicted sports injury incidence (including concussion). In line with our preregistration, we opted for odds ratios to minimise the potential for error due to conversion between effect sizes, and because most papers reported odds ratios to examine this relationship. For this first meta-analysis, we focused on injury incidence as it provided a standardised binary metric (injured vs non-injured), unlike injury severity and duration, which were defined inconsistently across studies. For example, studies measured injury severity continuously as a symptom sum score, or categorically as a clinical diagnosis or loss of consciousness. Injury duration was likewise measured in varying metrics (e.g. days, weeks, months or even years for chronic injuries). The second meta-analysis investigated whether the sports-related concussion incidence predicted worse mental health and well-being. The third meta-analysis investigated whether other sports injury incidence predicted worse mental health and well-being. For these two meta-analyses, we extracted quantitative measures of means and standard deviations for mental health and well-being outcomes, focusing on studies with control groups (i.e. to allow the comparison of injured vs non-injured participants). Our preregistration specified Cohen’s d as the preferred effect size. However, several studies reported associations between binary injury status and continuous mental health or well-being outcomes as correlations (point-biserial or Pearson’s r), which we transformed to Fisher’s *z* for a meta-analysis. This approach stabilises variance and enables reliable pooling of correlations, following standard practice for meta-analyses of this type. Study weights in the random-effects model were calculated as the inverse of the variance of each study’s Fisher z-transformed correlation, so that more precise studies contributed greater weight to the pooled estimate [23].

Analyses were conducted in R (4.1.2) with the meta package [24]. Given considerable between-study heterogeneity, we used an inverse variance random-effects model to pool effect sizes. We assessed study variability with the I^2^ estimate of heterogeneity and calculated the heterogeneity variance τ^2^ with the restricted maximum likelihood estimator [25]. To control for uncertainty in our estimate of between-study heterogeneity, we applied Knapp–Hartung adjustments to calculate the confidence interval around the pooled effect [26].

## Results

### Description of Included Studies

**Fig. 1:**
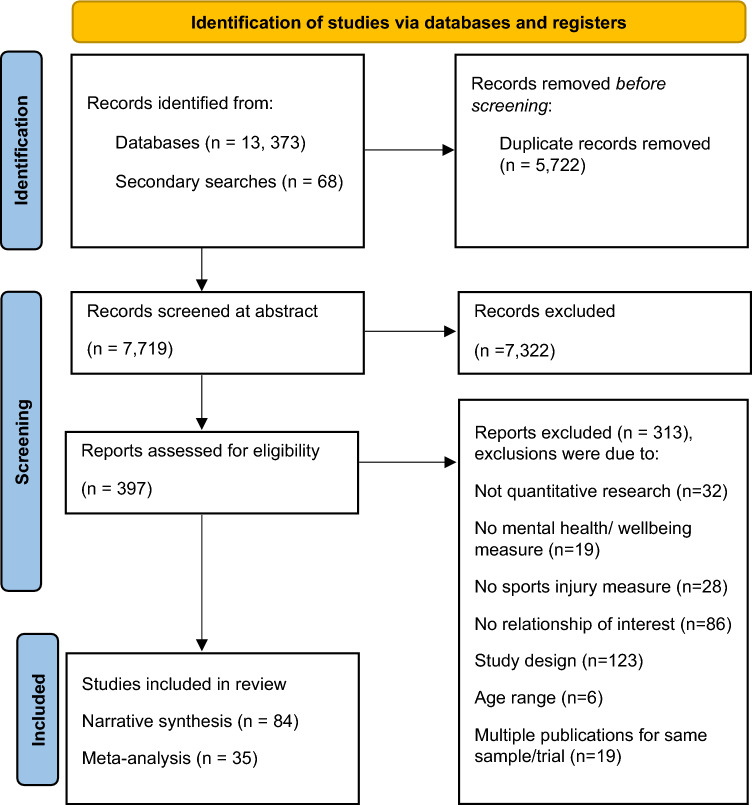
PRISMA (Preferred Reporting Items for Systematic reviews and Meta-Analyses) flow diagram depicting the identification and screening process of included studies

We identified 13,441 records through database searching. After deduplication, we screened 7719 abstracts and read 397 articles in full. Three hundred and thirteen studies were excluded as they did not meet the inclusion criteria, and we retained a total of 84 studies for the systematic review (Fig. 1).

In total, 221,095 adolescents and young people (mean age 10–24 years, range 0–29 years) were represented in this systematic review. Sixty-seven percent of papers (*n* = 56) focused on concussions or cumulative head impact exposure. After concussions, the most common types of sports injuries included were knee, ankle and musculoskeletal injuries. Measures of mental health included scales assessing depression, anxiety, suicide ideation/attempts and psychiatric diagnoses (e.g. attention-deficit hyperactivity disorder), while well-being measures included QOL, happiness and life satisfaction. We note that most papers did not report data disaggregated by age or sex, and hence we only report these descriptively. A summary of study characteristics for the 84 included studies is provided in the ESM.

Almost two thirds (65%) of the studies were from the USA (*n* = 55) and approximately 14% of the studies were from Canada (*n* = 12), followed by Australia (*n* = 5), Sweden (*n* = 3), UK, France, Norway, Spain, Finland, Turkey, Bosnia and Herzegovina, Taiwan and South Korea (each *n* = 1). A broad range of sports were represented from the grass roots to elite level. Studies followed athletes from club sports and “backyard” sports [27], to secondary school football, basketball, baseball, lacrosse and cheerleading [28, 29]. Studies also followed university athletes playing American Football at the national level [30, 31], hockey players in the elite league [32, 33], elite rugby league players in the National Rugby League [34], competitive and elite-level gymnasts (i.e. those participating in national or international competitions [35]), as well as track and field athletes qualified for world championships [36].

In terms of study design, 69% of studies were longitudinal (*n* = 58), and the rest were cross-sectional (*n* = 26). Most studies included a control group (*n* = 50) of non-injured or differently injured (e.g. concussion vs combined musculoskeletal injury) participants. We identified six intervention studies [27, 37–41], three of which were RCTs [38–40]. These studies provided evidence that rehabilitations targeting sports-related concussions significantly improved mental health and well-being outcomes (e.g. depression, fatigue and QOL). However, as interventions can impact multiple outcomes independently, such findings do not confirm that concussion directly causes mental health difficulties.

Thirty-four percent of studies (*n* = 29) assessed the bidirectional relationship between sports injuries and mental health/well-being. Twenty-three percent of studies (*n* = 19) investigated the impact of mental health and well-being on sports injuries. Forty-three percent of studies (*n* = 36) investigated the impact of sports injuries on mental health and well-being.

In terms of risk of bias, the majority of papers were found to be of overall fair (medium) quality, containing some concerns (ESM). Only three papers (which were RCTs) were preregistered. The most common quality issues included a lack of justification for sample size or power calculation, insufficient information to allow for independent replication, unclear motivation for measurement tools and the use of measures that were not validated.

### What is the Impact of Mental Health and Well-Being on Combined Sports Injuries?

Most studies that considered different types of sports-related injuries collectively found that pre-existing mental health and well-being problems were associated with an increased risk of sports injury incidence, severity, and prolonged symptom duration or recovery [28, 41–64].

### Meta-analysis of Mental Health/Well-Being Predicting Sports Injury Incidence

**Fig. 2:**
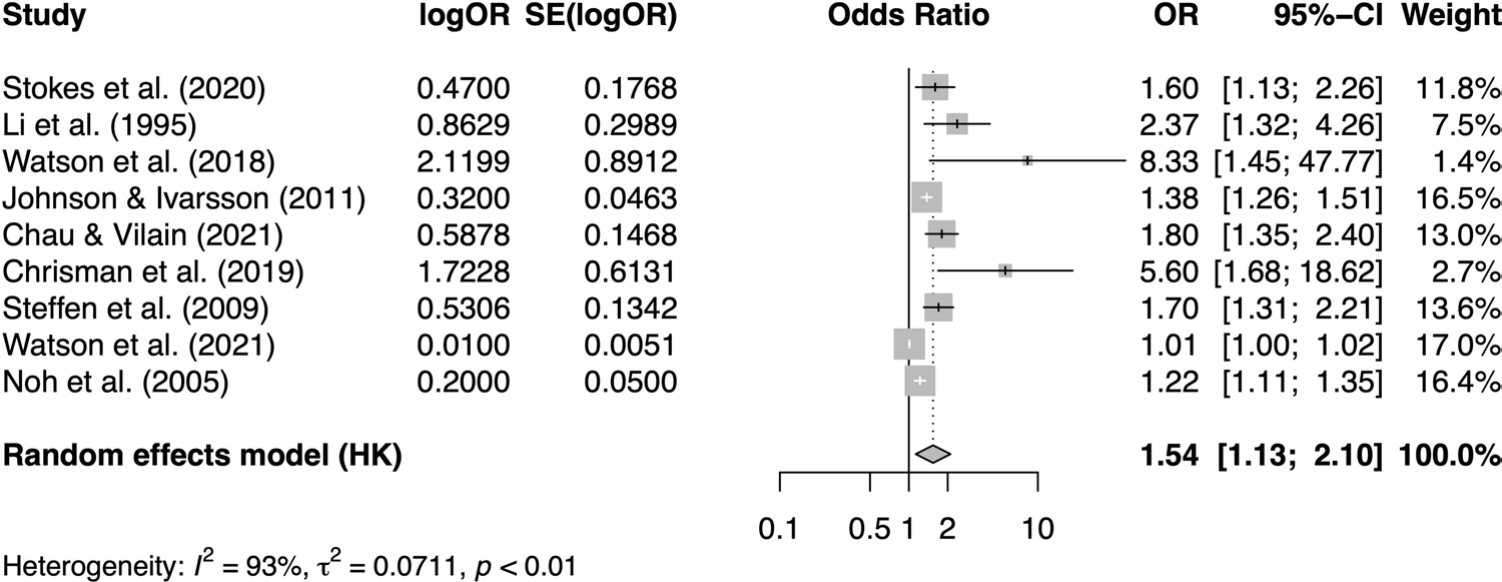
Forest plot of studies examining mental health/well-being as a predictor and sports injury incidence as an outcome [42, 45, 46, 48, 51, 54, 55, 64, 65]. *CI* confidence interval, *HK* Hartung–Knapp, *OR* odds ratio, *SE* standard error

We were able to meta-analyse nine studies that examined mental health or well-being as a predictor and sports injury incidence as an outcome. Our random-effects model indicated that worse mental health or well-being was associated with a significantly increased risk of sports injury incidence (odds ratio = 1.54, 95% confidence interval [CI] 1.13, 2.10, *p* < 0.01; *I*^2^ = 93%, *τ*^2^ = 0.07; see Fig. 2).

Our funnel plot (ESM) and Egger’s test (*p* < 0.01) indicated the presence of asymmetry, suggesting potential publication bias. To ascertain the magnitude of publication bias, we conducted the Duval and Tweedie trim-and-fill procedure [66], imputing five studies (ESM) and pooling the imputed effect sizes that yielded an overall effect that was no longer significant (odds ratio = 1.10, 95% CI 0.63, 1.91, *p* = 0.72; *I*^2^ = 92%, *τ*^2^ = 0.57).

### What is the Impact of Mental Health and Well-Being on Concussion-Related Sports Injuries?

Higher levels of mental health and well-being difficulties, specifically depression, anxiety and sleep disturbance, were found to predict a higher incidence and severity of concussion, longer recovery times and persistence of post-concussive symptoms in youth athletes compared with those with fewer mental health challenges [28, 41, 43–45, 49, 50, 52, 53, 56–59, 61, 63, 64, 67]. For example, US secondary school students who had seriously considered or attempted suicide were more likely to have experienced a sports-related concussion compared with their peers without suicidal ideation [63]. Interestingly, antidepressant use was found to interact with premorbid depression and anxiety to influence concussion; a longitudinal study of youth athletes in the USA found that premorbid depression or anxiety was associated with an increased risk of concussion incidence and prolonged recovery amongst athletes who were taking antidepressants, but not athletes who were unmedicated [67]. Children with pre-existing depressive and anxiety symptoms displayed significantly prolonged symptom duration or recovery from concussion [44, 50, 56], whilst those with prior psychiatric diagnoses, in particular attention-deficit hyperactivity disorder, depression and anxiety disorder, were also more likely to experience post-concussion syndrome, or prolonged recovery from concussion [41, 43, 49, 53].

### What is the Impact of Mental Health and Well-Being on Other Musculoskeletal Sports Injuries?

When limiting consideration to musculoskeletal injuries (excluding concussion), the majority of studies indicated that pre-existing mental health and well-being issues were associated with increased risk, severity and duration of sports injury symptoms [42, 46–48, 51, 54, 55, 60, 62]. For example, Swedish professional and junior soccer players with higher trait anxiety and life event stress were at an increased risk of sustaining a sports injury later in the season [47, 48]. French adolescent athletes with depressive symptoms were at a significantly higher risk of incurring sports and training injuries, even after controlling for socioeconomic status, obesity, alcohol and tobacco use and poor health status [51]. In young Australian adults, self-harm predicted injury incidence [42]. Conversely, higher well-being in the preceding week significantly reduced subsequent injury severity among elite adolescent athletes participating in athletics, skiing and handball [60]. Among ballerinas, those with lower levels of confidence and increased worry were significantly more likely to experience a ballet injury; moreover, freedom from worry and negative dance stress were significant predictors of injury duration [55].

However, not all studies found that prior mental health and well-being influenced sports injury incidence, severity and duration [29, 32, 67–72]. In some cases, there were mixed findings as other psychosocial factors came into play. Amongst Canadian elite ice hockey athletes, competitive state anxiety and fear of reinjury did not predict sports injury incidence; however, weak athletic identity was a risk factor for a first-time injury, and strong athletic identity significantly predicted the risk of subsequent injury [32]. Similarly, while there was no association found between pre-season competitive anxiety and life stress with injury time loss amongst elite Australian athletes, pre-season optimism and hardiness were associated with decreased injury time loss [71]. These psychosocial factors are reviewed in greater detail in Sect. 3.10.

### What is the Impact of Sports-Related Concussion on Mental Health and Well-Being?

Studies consistently demonstrated that sports-related concussions predicted significantly higher depression severity and anxiety as well as irritability, emotional stress, frustration and fatigue [56, 73–76]. Higher severity or prolonged duration of post-concussion symptoms was associated with greater impairments in mental health and well-being, particularly in the weeks and months following injury. Rehabilitation interventions targeting sports-related concussions were generally associated with improvements in depression and anxiety symptoms, mood, fatigue and health-related QOL [27, 37–41]. While these findings suggest that concussions may contribute to poorer mental health and well-being, we caution that intervention effects could operate through multiple pathways and therefore cannot be interpreted as evidence of causality.

A minority of studies did not find a negative impact of sports-related concussions on mental health and well-being [68, 69, 77, 78]. However, some of them found that concussions did impact other areas of health and cognition, which we briefly outline as they are beyond the scope of this review. For example, US university athletes who experienced concussions reported significantly worse sleep disturbance than healthy controls [77], while Canadian adolescents with concussions displayed significantly impaired working memory accuracy compared with controls [78].

### Meta-Analysis of Concussion Incidence Predicting Mental Health/Well-Being

**Fig. 3:**
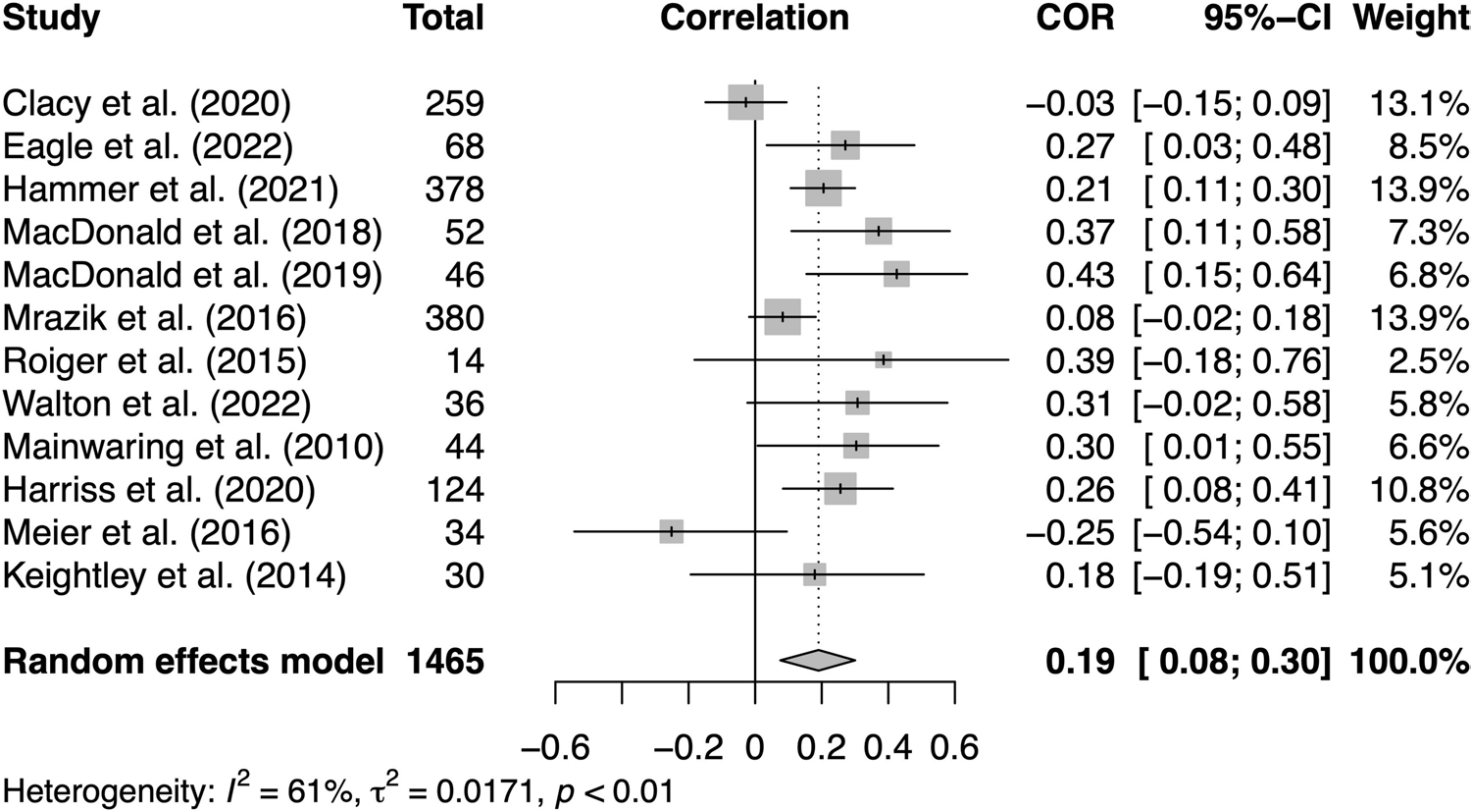
Forest plot of studies examining concussion incidence as a predictor and mental health/well-being as an outcome [30, 31, 33, 68, 70, 74, 77–82]. *CI* confidence interval, *COR* correlation coefficient

We were able to meta-analyse 12 studies that examined concussion incidence as a predictor and mental health or well-being as an outcome, with a control group that allowed us to compare the mental health/well-being symptoms of concussed versus non-concussed participants. Our random-effects model indicated that concussion incidence was significantly associated with worse mental health or well-being (*Z*_*r*_ = 0.19, 95% CI 0.08, 0.30, *p* < 0.01; *I*^2^ = 61%, *τ*^2^ = 0.02; see Fig. 3).

Our funnel plot (ESM) and Egger’s test (*p* = 0.21) did not indicate the presence of asymmetry, suggesting that publication bias was not likely. We conducted the trim-and-fill procedure as a sensitivity test, which imputed three studies (ESM), and pooling the imputed effect sizes yielded an overall effect that remained significant (*Z*_*r*_ = 0.14, 95% CI 0.02, 0.26, *p* = < 0.05; *I*^2^ = 63%, *τ*^2^ = 0.02).

### What is the Impact of Other Musculoskeletal Sports Injuries on Mental Health and Well-Being?

We found that most studies demonstrated that adolescents who experienced a sports injury experienced worse mental health and well-being [31, 33, 34, 56, 62, 65, 73–76, 80, 81, 83–96]. Amongst US competitive university athletes, injured athletes displayed significantly higher self-reported depression symptoms than non-injured controls [89, 90]. Moreover, this pattern persisted over time, as clinician-rated depression symptoms for injured athletes remained elevated above non-injured controls for up to 1-month post-injury [89]. British professional soccer players under 23 years of age who experienced a sports injury displayed significantly worse mental well-being than their uninjured peers [91]. Notably, the length of time spent injured accounted for the most variance in well-being, highlighting the important role of injury duration in influencing soccer athletes’ well-being.

Sports injuries were also associated with significantly lower QOL in US, Canadian and Turkish samples [65, 92–96]. The impact of knee and ankle injuries on QOL appeared to be particularly chronic, as Canadian youth athletes who had a history of sports-related knee injuries still demonstrated worse condition-specific health-related QOL 3–12 years later [95], while those who had experienced a time-loss ankle sprain reported decreased QOL, poorer self-reported function and greater fear of pain 3–15 years later [96]. There was also a dose–response relationship regarding injury severity and QOL. For instance, a US sample of injured female volleyball athletes demonstrated worse QOL than their uninjured peers, and those with a season-ending injury showed a significantly greater decrease in QOL than their less severely injured peers who could return to play during the season [65].

### Meta-analysis of Sports Injury Incidence (Excluding Concussion) Predicting Mental Health/Well-Being

**Fig. 4:**
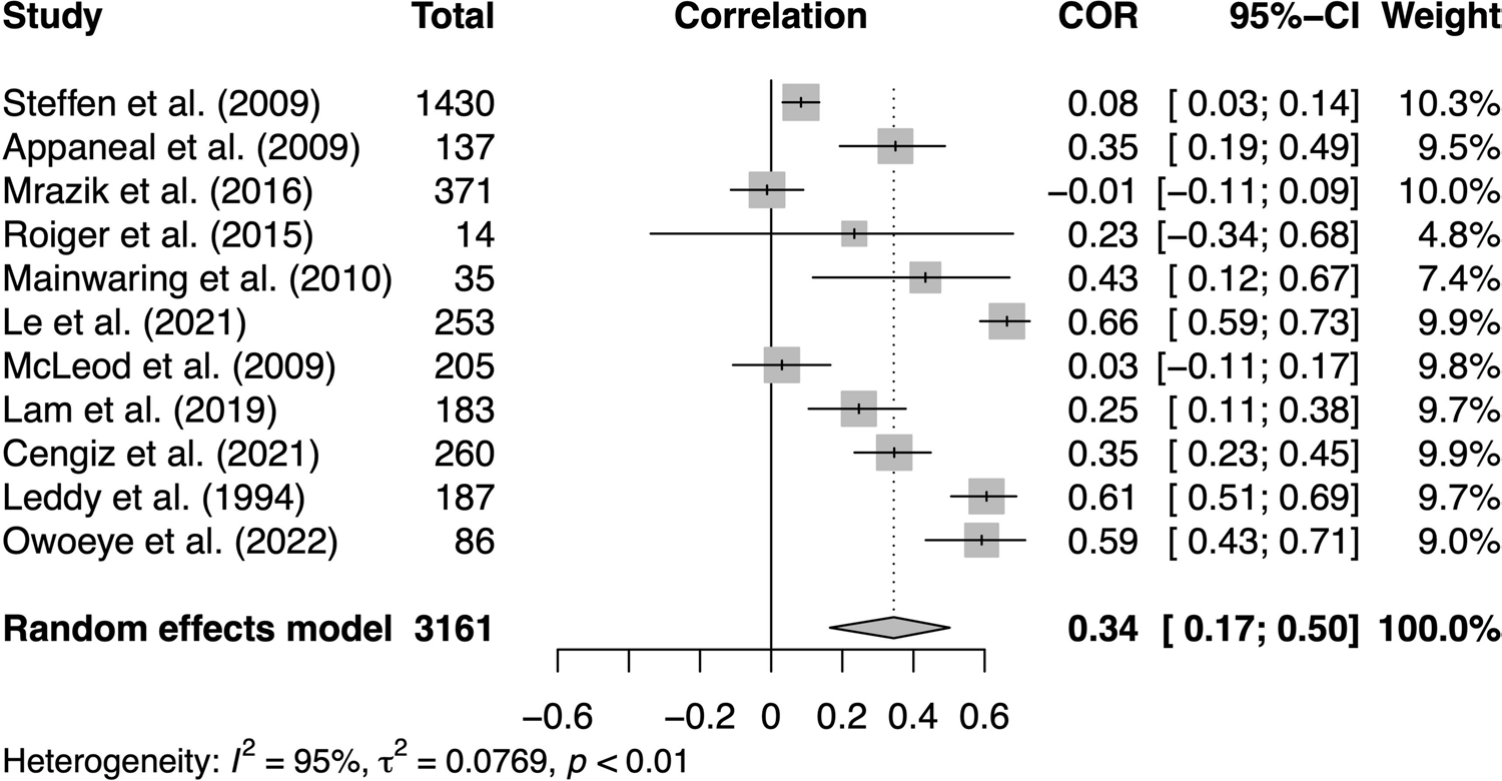
Forest plot of studies examining sports injury incidence as a predictor and mental health/well-being as an outcome [31, 33, 54, 81, 86, 89, 90, 92, 94–96]. *CI* confidence interval, *COR* correlation coefficient

We were able to meta-analyse 11 studies that examined sports injury incidence (excluding concussion) as a predictor and mental health or well-being as an outcome, with a control group that allowed us to compare the mental health/well-being symptoms of injured versus non-injured participants. Our random-effects model indicated that sports injury incidence was significantly associated with worse mental health or well-being (*Z*_*r*_ = 0.34, 95% CI 0.17, 0.50, *p* < 0.01; *I*^2^ = 95%, *τ*^2^ = 0.08; see Fig. 4).

Our funnel plot (ESM) and Egger’s test (*p* = 0.11) did not indicate the presence of asymmetry, suggesting that publication bias was not likely. We implemented the trim-and-fill procedure as a sensitivity test, which imputed five studies (ESM), and pooling the imputed effect sizes yielded an overall effect that was no longer significant (*Z*_*r*_ = 0.12, 95% CI − 0.11, 0.34, *p* = 0.27; *I*^2^ = 97%, *τ*^2^ = 0.18).

### What are the Potential Mechanisms Influencing the Relationship Between Overall Sports Injuries and Mental Health/Well-Being?

Several longitudinal and cross-sectional studies suggest that psychosocial factors may shape how injuries affect mental health outcomes in young athletes. For example, a US longitudinal study of injured adolescent athletes found that low positive stress (i.e. perceiving stressors as positive) and an increased sense of athletic identity predicted higher post-injury depressive symptoms after controlling for injury severity and sex [97]. Additionally, injured adolescents who received higher social support demonstrated decreased depressive symptoms, although neither social support nor coping moderated the relationship between injury severity and depression.

Similarly, in a Taiwanese sample of injured university athletes, social support and hope predicted athletes’ rehabilitation beliefs and well-being with an interactive effect: for athletes with low hope, social support was associated with higher levels of well-being, whereas social support demonstrated a lower association with well-being amongst athletes with high hope [98]. Other individual factors also appear to play an important role. Among US youth athletes who had sustained concussions, those who displayed high athletic identity, performance anxiety and a lack of motivation were more likely to experience prolonged post-concussion symptomatology and slower recovery [58]. In Australian youth athletes competing at the state, national or international level, personality traits moderated the relationship between injury time loss and stress [71]. Specifically, optimism and hardiness were associated with decreased injury time loss when positive life change increased, while self-esteem was associated with decreased injury time loss when both negative and total life change increased.

Taken together, these studies suggest that psychosocial factors such as stress appraisals, athletic identity, social support, performance anxiety and personality traits can influence how sports injuries impact mental health in young athletes. Some factors (e.g. social support, optimism, hardiness, self-esteem) appear to buffer against negative psychological outcomes, whereas others (e.g. strong athletic identity, performance anxiety, lack of motivation) may exacerbate distress or prolong recovery [58, 71, 97, 98]. These findings provide preliminary evidence that psychosocial characteristics may act as potential mechanisms in the sports injury-mental health relationship, but further research is needed to confirm and clarify these pathways.

## Discussion

In this systematic review and meta-analysis of 221,095 adolescents engaging in sports, we found significant evidence for a bidirectional relationship between sports injuries and mental health. Sports-related concussion and other sports injuries were associated with worse mental health (e.g. depression, anxiety) and well-being (e.g. stress, QOL) compared with controls. Conversely, adolescent athletes with poor mental health and well-being were approximately 1.5 times more likely to sustain an injury compared with their peers without mental health problems; however, adjusting for publication bias rendered this association non-significant, highlighting the need for caution when interpreting this particular result.

Given the high rates of sports participation and specific risk factors for injury in this demographic [7, 9], as well as the increasing concern about the mental health and well-being of adolescents worldwide [99], this complex two-way relationship warrants greater attention. A recent clinical review characterised this relationship as a “vicious cycle” in which poor mental health exacerbates the risk of sports injury and injury exacerbates the risk of poor mental health [14]. Although researchers have proposed theories such as the stress-injury model to explain why this reciprocal relationship occurs [100], our review highlights a notable lack of direct empirical evidence identifying specific mechanisms that can be confidently targeted in interventions. However, preliminary evidence does suggest that psychosocial factors (such as athletic identity and social support) may be important and modifiable contributors. Therefore, interventions to support long-term sports participation and mental health are warranted, but recommendations currently rely on promising but still developing evidence [101, 102].

We found a small number of studies focusing on psychosocial factors (e.g. athletic identity, social support) that may be effective targets in interventions aiming to ameliorate mental health pre- or post-injury. A recent meta-analysis of 11 RCTs and intervention control trials found that preventive psychological interventions moderately reduced sports injury incidence, particularly those targeting cognitive behaviour [103]. However, because of substantial heterogeneity in the intervention type across studies (e.g. stress management, cognitive behavioural therapy, attention training), there is yet to be a consensus regarding the “gold standard” psychological approach to reducing sports injury. Additionally, emerging studies indicate that mindfulness interventions might reduce injury risk amongst adolescent athletes [104, 105], although further research is required as the current evidence base for mindfulness is still nascent [106]. To date, we are not aware of any intervention studies that target athletic identity. Considering the importance of peer influence during adolescence, experiencing a sports injury might not only threaten the young athlete’s sense of identity, but also remove them from their main social network [11]. Thus, future interventions could teach adolescent athletes to expand their identity independently of their athletic ability (e.g. focusing on core values), and provide them with tools to derive social support from family members and other peer groups outside of sport.

### Strengths and Limitations

To our knowledge, this is the first systematic review to quantitatively examine the bidirectional relationship between sports injuries and mental health in adolescent athletes. A strength of our study is the broad inclusion criteria, encompassing various types of sports-related injuries and a wide range of mental health outcomes, from clinical diagnoses to measures of poor well-being. By focusing on adolescents, the study addresses a critical developmental period for the emergence of mental health issues. However, several limitations were identified, including a lack of consistency in measuring instruments across studies. The heterogeneity in how sports injuries and mental health outcomes were reported, including the variability in timing of assessments relative to injury, precluded the meta-analysis of most extracted data and limited the extent to which we could synthesise findings. A key limitation of much of the included evidence is the reliance on cross-sectional designs, which preclude conclusions about the directionality of the relationship between sports injury and mental health. In such studies, differences in mental health around the time of injury could reflect pre-existing conditions that both raise injury risk and worsen after injury (i.e. confounding). Where available, we prioritised the interpretation of findings from longitudinal and interventional studies to mitigate these biases. Although our systematic review aimed to include studies from across the world, most research originated from high-income settings, predominantly the USA and Canada. Strikingly, we identified only one study from the UK [91], highlighting the scarcity of UK-based research. We identified only two studies from East Asia [55, 98], and no studies from low- or middle-income countries. Given that the majority of the world’s adolescents reside in low- or middle-income countries, there is a clear need for research from more diverse settings.

### Implications for Future Research and Practice

Our review demonstrated potential publication bias in existing studies. Notably, only three RCTs were preregistered. More robust, preregistered longitudinal studies are needed, spanning at least a season to monitor how an injured athlete’s mental health is impacted over time. Longitudinal designs should also aim to elucidate causal pathways and mechanisms, taking a multi-perspective lens that considers biological, psychological and social factors. Researchers should more clearly report the timing of mental health measurements in relation to injury occurrence (e.g. pre- and post-injury) and the sports season (e.g. an injury at the end of the season might impact mental health less severely than at the start). Our findings underscore the need for rigorous research practices and reporting of non-significant results, as we found evidence of publication bias in one of our three meta-analyses. We also call for more participatory and co-produced studies, as collaborating with athletes who have lived experience of sports injuries and mental health difficulties is necessary to advance the quality and validity of research. We acknowledge that coaches and clubs may already be aware of the benefits of monitoring athletes’ mental health. We urge the wider adoption of this practice for all young athletes, and see this as a key policy recommendation. We provide a summary of both research and policy implications in Table 1. Our findings align with the *International Olympic Committee’s Mental Health Surveillance Supplement*, which calls for longitudinal mental health surveillance monitoring throughout the entire season [107]. We extend these points by emphasising the need for an explicit focus on young athletes, which incorporates a consideration of psychosocial factors that may influence the relationship between sports injury and mental health. We recommend that well-validated mental health measures should be integrated with existing injury surveillance systems for young athletes to support a more holistic approach. There is an increasing awareness among coaches, parents and young people about the importance of mental health in sports, yet the complex reciprocal relationship between mental health and sports injuries is less well known. As such, there is a growing call for more integrated approaches that address both physical and mental aspects of sports injuries among youth. We substantiate the call for mental health clinicians to be integrated into interdisciplinary sports medicine teams, preferably professionals who possess specific knowledge of athletic culture [14]. Finally, tailoring early interventions specifically towards adolescent athletes and targeting psychosocial mechanisms that particularly impact adolescents (e.g. developing a non-athletic identity and peer support outside of sport, or improving executive control over impulsivity and risk-taking tendencies) could help to improve injury risk and recovery rates, facilitate return-to-play, build resilience and promote long-term participation overall.

**Table 1:** Summary of research and policy implications and recommendations

Research implications and recommendations	• To better understand the relationship between sports injury and mental health in young athletes, there is a need for research on mechanisms and causality, including: o Need for longitudinal data to clarify temporal relationships between sports injury and mental health o Need for RCTs of psychosocial interventions (e.g. stress management, social support programmes) for decreasing injury risk and improving post-injury mental health • There is a need for better measurement and stronger empirical designs, including: o Need for preregistered studies to enhance transparency, reduce risk of selective reporting and promote open science practices o Need for greater inclusion and consideration of mental health o Need for precise measures that do not contain items that relate to both injury and mental health (e.g. the Post-Concussion Symptom Scale is used as a concussion severity scale and also contains measures for emotional impact) • There is a need for stronger reporting standards, including: o Clearly reporting timing of measurements relative to season (e.g. pre-season, during or post-season) o Clearly reporting timing of the mental health assessment relative to occurrence of injury (e.g. pre-, at the same time or post-injury) o Improved reporting of injury type, and injury severity (e.g. minor, major, season or career ending) o Including assessments of how and why injury occurred (e.g. lack of protective gear, intensity, accident) o Clearly justifying reasons for measurement choice o Transparently reporting measures to allow future replication and comparison between studies (e.g. concussion should be measured consistently across studies) • Need for a focus on understanding lived experience o Emphasise participatory and co-produced research approaches to ensure young athletes’ voices shape study design, measures and interpretation • Expand research beyond the Global North o Current research largely stems from the Global North; thus, broader geographic and cultural coverage is needed to capture the diverse athlete experiences across different sports and sociocultural contexts
Policy implications and recommendations	• A young athlete’s mental health should be considered as part of their holistic evaluation in terms of performance ability, injury risk and long-term athletic activities o This can include incorporating mental health screenings into routine annual evaluations, identifying early signs of psychological distress and providing access to mental health support • Mental health assessment should be incorporated into “return to play” evaluations and considerations • Coaches, training and support staff should be trained to recognise the importance of mental health and its bidirectional links with sports injury • There is a need to strengthen and develop UK-specific policy guidance to address the current lack of research on the links between athlete mental health and injury in the UK

*RCT* randomised controlled trial

## Conclusions

This systematic review and meta-analysis provides significant evidence for a bidirectional relationship between sports injuries and mental health in adolescent athletes. Future research should focus on investigating the mechanisms underlying this relationship to inform early interventions, as well as applying preregistered, longitudinal and co-produced designs. Mental health assessments, support and education should become integral components of sports medicine practices to ensure holistic care and improve both physical and psychological health outcomes for athletes.

## Supplementary Information

Below is the link to the electronic supplementary material.

Supplementary file1 (PDF 864 KB)

## Declarations

### Availability of data and material

Data come from published papers that are publicly available online.

### Code availability

The statistical code will be made available on reasonable request to the corresponding author.

### Patient and public involvement

At the conceptualisation and interpretation stages of this project, we engaged with experts in sports and mental health to leverage their expertise and experience working with young athletes. We have presented our findings at sports science events and conferences at the University of Oxford and Podium Institute, which were attended by athletes, sports representatives and members of the public, and we were able to use their feedback for this manuscript. To disseminate our findings to the public, we aim to write social media posts and blog posts outlining the main conclusions of our paper (e.g. on the websites of the University of Oxford’s Department of Experimental Psychology and the Podium Institute for Sports Medicine and Technology).
