# Supplementary material for: Bidirectional Relationship Between Mental Health and Sports Injury in Adolescents: A Systematic Review and Meta-analysis
Source: Sports Med. 2026 Jan 13;56(4):981–95. doi: 10.1007/s40279-025-02379-z (PMC13124830; doi:10.1007/s40279-025-02379-z)

Bidirectional Relationship Between Mental Health and Sports Injury in Adolescents: A Systematic Review and Meta-analysis

Sports Medicine

Supplementary Material

Athena R. W. Chow[1], BSc; Mirela Zaneva[1,2], PhD; Layla Rashid[1], MSc; Catherine Wheatley[3], PhD; Constantin Coussios[4], PhD; Robert Hepach[1], PhD; Lucy Bowes[1], PhD

Author affiliations

[1] Department of Experimental Psychology, University of Oxford, Oxford, UK.

[2] Christ Church College, University of Oxford, UK.

[3] Nuffield Department of Clinical Neurosciences, University of Oxford, UK.

[4] Institute of Biomedical Engineering, Department of Engineering Science, University of Oxford, UK.

Corresponding author: Athena Chow. Email: athena.chow@magd.ox.ac.uk

Supplementary Material 1. PRISMA Checklist

Section and Topic	Item #	Checklist item	Location where item is reported
**TITLE**			
Title	1	Identify the report as a systematic review.	1
**ABSTRACT**			
Abstract	2	See the PRISMA 2020 for Abstracts checklist.	2
**INTRODUCTION**			
Rationale	3	Describe the rationale for the review in the context of existing knowledge.	3
Objectives	4	Provide an explicit statement of the objective(s) or question(s) the review addresses.	3-4
**METHODS**			
Eligibility criteria	5	Specify the inclusion and exclusion criteria for the review and how studies were grouped for the syntheses.	4
Information sources	6	Specify all databases, registers, websites, organisations, reference lists and other sources searched or consulted to identify studies. Specify the date when each source was last searched or consulted.	4
Search strategy	7	Present the full search strategies for all databases, registers and websites, including any filters and limits used.	Supplement 2
Selection process	8	Specify the methods used to decide whether a study met the inclusion criteria of the review, including how many reviewers screened each record and each report retrieved, whether they worked independently, and if applicable, details of automation tools used in the process.	5
Data collection process	9	Specify the methods used to collect data from reports, including how many reviewers collected data from each report, whether they worked independently, any processes for obtaining or confirming data from study investigators, and if applicable, details of automation tools used in the process.	5
Data items	10a	List and define all outcomes for which data were sought. Specify whether all results that were compatible with each outcome domain in each study were sought (e.g. for all measures, time points, analyses), and if not, the methods used to decide which results to collect.	5
	10b	List and define all other variables for which data were sought (e.g. participant and intervention characteristics, funding sources). Describe any assumptions made about any missing or unclear information.	5
Study risk of bias assessment	11	Specify the methods used to assess risk of bias in the included studies, including details of the tool(s) used, how many reviewers assessed each study and whether they worked independently, and if applicable, details of automation tools used in the process.	5
Effect measures	12	Specify for each outcome the effect measure(s) (e.g. risk ratio, mean difference) used in the synthesis or presentation of results.	5-6
Synthesis methods	13a	Describe the processes used to decide which studies were eligible for each synthesis (e.g. tabulating the study intervention characteristics and comparing against the planned groups for each synthesis (item #5)).	5-6

Section and Topic	Item #	Checklist item	Location where item is reported
	13b	Describe any methods required to prepare the data for presentation or synthesis, such as handling of missing summary statistics, or data conversions.	5-6
	13c	Describe any methods used to tabulate or visually display results of individual studies and syntheses.	5-6
	13d	Describe any methods used to synthesize results and provide a rationale for the choice(s). If meta-analysis was performed, describe the model(s), method(s) to identify the presence and extent of statistical heterogeneity, and software package(s) used.	6
	13e	Describe any methods used to explore possible causes of heterogeneity among study results (e.g. subgroup analysis, meta-regression).	6
	13f	Describe any sensitivity analyses conducted to assess robustness of the synthesized results.	6
Reporting bias assessment	14	Describe any methods used to assess risk of bias due to missing results in a synthesis (arising from reporting biases).	5
Certainty assessment	15	Describe any methods used to assess certainty (or confidence) in the body of evidence for an outcome.	5
**RESULTS**			
Study selection	16a	Describe the results of the search and selection process, from the number of records identified in the search to the number of studies included in the review, ideally using a flow diagram.	7
	16b	Cite studies that might appear to meet the inclusion criteria, but which were excluded, and explain why they were excluded.	7
Study characteristics	17	Cite each included study and present its characteristics.	7-17, Supplement 3
Risk of bias in studies	18	Present assessments of risk of bias for each included study.	8 (as a whole)
Results of individual studies	19	For all outcomes, present, for each study: (a) summary statistics for each group (where appropriate) and (b) an effect estimate and its precision (e.g. confidence/credible interval), ideally using structured tables or plots.	8-16
Results of syntheses	20a	For each synthesis, briefly summarise the characteristics and risk of bias among contributing studies.	8-16, Discussion
	20b	Present results of all statistical syntheses conducted. If meta-analysis was done, present for each the summary estimate and its precision (e.g. confidence/credible interval) and measures of statistical heterogeneity. If comparing groups, describe the direction of the effect.	10-16
	20c	Present results of all investigations of possible causes of heterogeneity among study results.	10-16
	20d	Present results of all sensitivity analyses conducted to assess the robustness of the synthesized results.	10-16
Reporting biases	21	Present assessments of risk of bias due to missing results (arising from reporting biases) for each synthesis assessed.	8, Supplement

Section and Topic	Item #	Checklist item	Location where item is reported
			4
Certainty of evidence	22	Present assessments of certainty (or confidence) in the body of evidence for each outcome assessed.	5, Discussion
**DISCUSSION**			
Discussion	23a	Provide a general interpretation of the results in the context of other evidence.	18-20
	23b	Discuss any limitations of the evidence included in the review.	19
	23c	Discuss any limitations of the review processes used.	19
	23d	Discuss implications of the results for practice, policy, and future research.	19-20
**OTHER INFORMATION**			
Registration and protocol	24a	Provide registration information for the review, including register name and registration number, or state that the review was not registered.	2-4
	24b	Indicate where the review protocol can be accessed, or state that a protocol was not prepared.	2-4
	24c	Describe and explain any amendments to information provided at registration or in the protocol.	6
Support	25	Describe sources of financial or non-financial support for the review, and the role of the funders or sponsors in the review.	17
Competing interests	26	Declare any competing interests of review authors.	17
Availability of data, code and other materials	27	Report which of the following are publicly available and where they can be found: template data collection forms; data extracted from included studies; data used for all analyses; analytic code; any other materials used in the review.	Supplement contains summary data extracted

From: Page MJ, McKenzie JE, Bossuyt PM, Boutron I, Hoffmann TC, Mulrow CD, et al. The PRISMA 2020 statement: an updated guideline for reporting systematic reviews. BMJ 2021;372:n71. doi: 10.1136/bmj.n71

Supplementary Material 2. Search Strategy

Search terms: (Age group) AND (sports) AND (injury) AND (mental health and wellbeing)

The same keywords will be used for each database (adapting for different syntax symbols if relevant) and searched in titles and abstract fields (or equivalent). We have translated syntax manually, against the guidelines for each databases and then also used the Polyglot tool to ensure we have correct syntax for each database https://sr-accelerator.com/#/polyglot

Our search strategy corresponding to the search terms above:

1. (adolescen* OR child* OR youth OR teen* OR boy* OR girl* OR 'young athlete' OR 'junior athlete' OR 'high school athlete')
2. (sport* OR athlet* OR 'physical activity' OR exercis* OR aerobic* OR 'physical fitness' OR running OR cycling OR bicycl* OR swim* OR basketball OR football OR soccer OR rugby OR 'rugby union' OR 'rugby league' OR trampoline OR skateboard or ski* OR snowboard* OR 'water polo' OR gymnast* OR 'field hockey' OR equestrian OR box* OR cricket)
3. (injur* OR sprain* OR strain* OR concuss* OR tear* OR damage* OR rupture* OR 'traumatic brain injury' OR tbi OR impinge* OR pain)
4. ('mental health' OR 'mental ill health' OR psych* funct* OR psych* health OR mental disorder* OR emotional problem* OR emotional funct* OR internali$ing OR anxi* OR depress* OR suicide* OR self* harm OR somati* OR withdraw* OR emotional functioning OR 'emotional symptom' OR 'emotional problem' OR emotional difficult* OR externali$ing OR behavio$ral problem* OR well$being OR behavio$ral funct* OR mental well$being OR psych* well$being OR happ* OR subjective well$being OR 'life satisfaction' OR 'quality of life')
5. Combine 1 AND 2 AND 3 AND 4
6. Apply filters for 1990-Current year; Human; search in title, abstract, key words; English

Supplementary Material 3. Table of Included Studies

**Author name(s) and year**	**N**	**Country**	**Age (mean; range)**	**Gender**	**Sport name**	**Type of injury**	**Type of mental health measure**	**Design**	**Descriptive summary of results**
Liberal et al. 2014	14	Spain	Mean 14.57 years - range 12-18	100% Female	Gymnastics	Not specified	1. Perceived psychological impact of an injury. 2. Psychological well-being.	Longitudinal, no control group, non-interventional	Results showed no relationship between psychological impact of injuries and injury duration.
Armento et al. 2021	90	US	13-18 (15.5 ± 1.2)	100% Female	Cheerleading/ Cross-country / Dance / Gymnastics / Marching band/ Pom / Soccer / Softball / Swimming / Tennis / Track and field / Unspecified / Volleyball / Basketball / Cheerleading / Cross-country / Equestrian / Gymnastics / Martial arts / Pom / Skiing / Soccer / Softball / Swimming / Track and field / Volleyball	Menstrual dysfunction: Examines presence of menstrual dysfunction - participants grouped if affirmative response to any of the following criteria: (1) age of menarche at ≥15 years of age, (2) three consecutive months without a menstrual period, or (3) ≤9 menstrual periods in the last 12 months.	Patient reported quality of life and health history	Cross-sectional, no control group, non-interventional	On univariable examination, those who were classified with menstrual dysfunction had higher fatigue and pain interference scores on the PROMIS questionnaires than those without menstrual dysfunction. After adjusting for age and BMI, menstrual dysfunction was significantly associated with higher anxiety, fatigue, and pain interference scores on the PROMIS questionnaires.
Lu & Hsu 2013	224	Taiwan	20.02 ± 1.47 years	53.57% Female	Track and field 20.54% Basketball 13.39% Volleyball 13.39%	Arm/ shoulder and hand (24.55%) Waist and back (13.39%)	Life satisfaction, positive affect and negative affect.	Cross-sectional, no control group,	No significant relationship was found between injury severity and

					Tae kwon do 12.5%<br>Chinese martial arts 8.93%<br>Judo 5.80%<br>Table tennis 5.36%<br>Other sports (Middle to long distance running, Tai chi, Soccer, Tennis, Dancing,<br>Rugby, Weight lifting) 20.09%	Leg (8.04%)<br>Ankle (25.89%)<br>Knee (15.63%)<br>Other injuries (12.50%)		non-interventional	subjective wellbeing, between injury severity and positive affect and between injury severity and negative affect.<br><br>Injury severity was not a predictor of subjective wellbeing in the hierarchal regression models.
Stokes et al. 2020	2154	Australia	20.99 (SD = 1.67)	54% female	Sport	Injury that required medical attention (e.g. bandaging, stitches, loss of a tooth, broken bones, or an amputation).	Self-harm	Longitudinal, no control group, non-interventional	Injury in 2012 was predicted by reported self-harm in the fully adjusted model but not the unadjusted model.
Rose et al. 2021	70	US	Mean (10.6 ± 0.64), range 9-12 years.	100% Male	Football	Cumulative head impacts	Previous medical diagnoses, including attention deficit/hyperactivity disorder (ADHD), anxiety, depression were recorded at the pre–season 1 visit. Parent report of ADHD-related symptoms of inattention and hyperactivity and Total difficulties subsection: self-reported difficulties with mood, behaviour,	Longitudinal, no control group, non-interventional	Repetitive head impacts in youth tackle football were not found to be associated with neurocognitive performance or behavioural outcomes.<br><br>Premorbid ADHD, depression or anxiety were not associated with concussion symptoms over time.<br><br>Previous concussions were not associated with

							and social adjustment.		SDQ or SWAN total scores.
Mac Donald et al. 2018	52	US	Control: 12.7 ± 1.6 Concussion: 13.0 ± 1.6 Range: 10-14	56% Female		Symptomatic concussion was defined as patients who had sustained a concussion during sports or recreational play that was diagnosed by a treating physician, whose symptoms had remained unresolved after a minimum of 4 weeks post-injury, and who had been seen in the Sports Medicine, Concussion, or Rehab Medicine specialty clinics at Seattle Children's Hospital.	1) Depression, 2) generalized anxiety disorder 3) sleep impairment 4) quality of life.	Cross-sectional, with control group, non-interventional	No statistically significant differences were found between concussed and control groups based on generalised anxiety disorder total severity score, anxiety symptoms impact life scores, has depressive symptoms scores, total PedsQL, getting along with others subscale, feeelings subscale score and adolescent sleep impairment measures. However there was a statistically significant difference between the groups for total depression severity scores and the school and health and activities scaled subscores of the quality of life measure.
Gagnon et al. 2016	10	Canada	Mean 16.3, range: 14-18	30% Female	Snowboarding (n=1), Hockey (n=2), Football (n=4), Soccer (n=2), Basketball (n=1)	Sport-related concussion	1) mood 2) energy level	Longitudinal, no control group, interventional	Depression scores, although within normal ranges, were significantly improved after

									intervention. Participants reported a significant decrease in all aspects of fatigue (general, cognitive, sleep/rest) after the intervention.
McCarty et al. 2021	200	US	Mean 14.7 [SD 1.7] years, range 11-18 years.	62% Female		Sports-related or recreational-related concussion<br><br>Concussion based on the (ICD-10) definition of post-concussion syndrome.	1)Depression 2) anxiety 3) suicide ideation 4) sleep quality 5) quality of life.	Longitudinal, with control group, interventional	Those who received collaborative care reported higher health-related quality of life at 12 months compared with a control group receiving usual care for adolescent report but not by parent report.<br><br>Sleep quality showed improvement for youth receiving collaborative care at all time points, compared with usual care and for all sleep subscales at 3 and 12 months compared with usual care.<br><br>No differences emerged by group over time for adolescent depressive or anxiety symptoms.

									The proportion of participants reporting suicidal ideation at the 12-month follow-up decreased significantly among adolescents receiving collaborative care compared with usual care.
Sarmiento et al. 2023	152266	US	Primarily high school students in grades 9 to 12. In some jurisdictions, grades 6 to 8 also participated.	Not reported	Played on a sports team (Yes)<br><br>Middle school 21.6% 95%CI(20.4-22.8)<br>High school 20% 95%CI(18.5-21.7)	Sports- or physical activity–related concussion during the 12 months before the survey. "A concussion is when a blow or a jolt to the head causes problems such as headaches, dizziness, being dazed or confused, difficulty remembering or concentrating, vomiting, blurred vision, or being knocked out."	Suicidality	Cross-sectional, no control group, non-interventional	Among both middle school and high school students, the prevalence of ≥1 sports- or physical activity–related concussions was higher among students who had seriously considered attempting suicide, had made a suicide plan, and had attempted suicide compared with those who had not engaged in those behaviours.
Eagle et al. 2022	782	US	12-25 years, n = 731/782 [94%] were 12-18 years old<br><br>Early cohort 15.4 ± 1.9 years.	51.7% Female		Sports-related concussion "a clear mechanism of injury and report of 1 or more concussion symptoms and/or signs (e.g., post-traumatic amnesia [PTA] or	History of psychiatric disorder (Anxiety and depression)	Longitudinal, no control group, non-interventional	Between-group comparisons found no significant differences between injury duration cohorts and history of ADHD/LD and psychiatric history.

			Middle cohort 15.8 ± 2.2 years. Late cohort 15.6 ± 1.8 years.			disorientation/confusion) following injury that were attributed to the head injury".			Both affective and sleep items in the PCSS were significantly greater in the Late group compared with the Early group. The Middle group did not have any significant mean differences compared with the Early group.
Howell et al. 2019	689	US	The median patient age was 15.3 years (range = 7.6-27.7 years)<br><br>Children (n= 137)<br>11.7 (10.7-12.4)<br><br>Adolescents (n= 478)<br>15.7 (14.4-16.9)	Children (7-12)<br>Female 26%<br>Adolescents (13-18)<br>Female 40%		Concussion was defined according to the latest International Consensus on Concussion in Sports. The working definition used was a brain injury caused by a direct blow to the head, face, neck, or elsewhere on the body, resulting in the rapid onset of impaired neurological function.	Sleep (feeling slowed down, drowsiness, fatigue/low energy, and trouble falling asleep), and emotional (more emotional than usual, irritable, sadness, and nervous/anxious)	Longitudinal, no control group, non-interventional	The emotional and sleep domains of the PCSS were not significantly associated with symptom duration.
McCarty et al. 2016	49	US	Mean 15.0 (SD 1.6)<br>Range (11-17) years	65% female		Sports- or recreation-related concussion.<br><br>A total of 57.5% of the adolescents incurred sports-related concussion, and 42.5% sustained recreation-related concussion (e.g.,	Depression, anxiety and quality of life.	Longitudinal, with control group, interventional	Adolescents assigned to collaborative care experienced clinically and statistically significant improvements in health-related quality of life

						being hit by a ball on the playground, trampoline injury).			demonstrated by both child and parent report. No significant improvement in depression for either parent or child report but 78% of intervention patients and 45.8% of control patients reported ≥50% reduction in depression symptoms. No significant changes between groups were demonstrated in anxiety symptoms for either parent or child report.
Howell et al. 2016	318	US	Median age for the cohort was 14.7 years (IQR = 12.9 -16.2).<br><br>Children (Age 8–12, n = 68) Median 11.7 years (IQR = 10.6 - 12.1)<br><br>Adolescents (Age 13–18, n = 250)	Children Female (21%) Adolescents Female (38%)	Ice hockey (21%), American football (20%), soccer (13%) and basketball (13%).	Concussion - a brain injury caused by a direct blow to the head, face, neck or elsewhere on the body, resulting in the rapid onset of impairment of neurological function.	Emotional symptoms (Emotionality, Irritability and sadness) and sleep symptoms (Feeling slowed down, Drowsiness, Fatigue or low energy, Trouble falling asleep)	Longitudinal, no control group, non-interventional	Variables that appeared different between the two groups with a statistical probability of $p < 0.20$ were identified as potential predictors and placed into logistic regression models. Emotional and sleep domains were therefore included for both children and adolescents as they differed between the two groups significantly.

			15.2 years (IQR = 14.2 - 16.8)						After adjusting for the effect of all included variables in a regression model, no independent association between sleep or emotional domains and consequent symptom duration was found among children or adolescents in the study.
Caze et al. 2021	40	US	13-14 years 45%<br>15-16 years 32.5%<br>17-18 years 25%	50% females		Concussion (50%) & Musculoskeletal (50%) controls	1) anxiety-related sensations (cognitive, physical and social)	Longitudinal, with control group, non-interventional	Concussed adolescents with higher anxiety sensitivity scores reported more initial symptoms than did those reporting musculoskeletal injury. Anxiety sensitivity was not related to the rate of symptom reduction over time for either group.
Chandler et al. 2022	236	US	Mean age = 14.3 ± 2.1 years; (range 8-18 years)	41% Female		A sport-related or recreation-related concussion.	Sleep disturbance, Fatigue, tiring more easily, Being irritable, easily angered, Feeling depressed or tearful, Feeling frustrated or impatient, Forgetfulness,	Longitudinal, no control group, non-interventional	Apart from the symptom of fear, participants reported symptoms of fatigue, frustration, irritability, taking longer to think, poor concentration, forgetfulness, emotional stress,

							poor memory, Reduced tolerance to stress or emotional excitement		sleep disturbance, restlessness and depression as significantly worse at 1 month follow up compared to pre-injury.<br><br>History of anxiety, depression, or previous head injury did not differ across the 2 symptom groups.
Mrazik et al. 2016	627	Canada	Concussed group (mean = 15.0 years; SD = 1.2)<br><br>Control group: (mean age = 14.93 years, SD = 1.2)<br><br>Range 12 - 17 years of age	16% Female	Hockey	Previous concussions and MSK injuries	Behaviour and self-perceptions, diagnosed medical and psychological conditions.	Cross-sectional, with control group, non-interventional	Results Indicated that a minority of participants with a history of either concussion alone or in combination with an MSK injury had higher ratings of psychological difficulties beyond normal recovery time frames. Athletes with a history of only MSK injuries without concussion did not present with lingering psychological outcomes compared with athletes with no history of injury.
Walton et al. 2022	40	US	Athletes: 19.3 ± 1.08 Controls:	Athletes: Females (55%)		Concussion	Anxiety, resilience, sleep disturbance,	Longitudinal, with control	Individuals with SC reported greater sleep disturbance

			20.8 ± 2.17 Range 18-29	Controls: Females (55%)			fatigue, and appetite	group, non-interventional	when compared with healthy controls. Moreover, only those with SC improved sleep, and fatigue over time. Anxiety and resilience improved in both groups throughout the study and feelings of hunger or fullness were not statistically different between groups or over time.
Gillie et al. 2022	129	US	(M = 14.4, SD = 2.3) range 10–18 years	49% Female		A concussion was defined per current guidelines and was diagnosed by clinicians trained in the assessment and treatment of concussions based on the following criteria: (1) clear mechanism of injury; (2) presence of one or more signs (e.g., loss of consciousness, posttraumatic amnesia, disorientation/confusion) and/or symptoms (e.g., headache, dizziness, nausea) at the time of injury; and (3) current symptoms and/or impairment (e.g.,	Anxiety	Cross-sectional, no control group, non-interventional	Total concussion symptom severity significantly predicted post injury mild anxiety symptoms but not clinical generalized anxiety disorders.

						cognitive, vestibular, ocular). Concussion was classified as either sport-related concussion (SRC) or non-SRC (e.g., assault, motor vehicle accident, fall).			
Stazyk et al. 2017	92	Canada	mean age of 15 years ± 2.5 range 7-18 years.	60.8% Female		Concussion (WHO diagnostic criteria).	Depression	Longitudinal, no control group, non-interventional	Children with evidence of depressive symptomatology had significantly higher mean post-concussive symptom inventory (PCSI) scores in recovery at initial visit and final visit than children who were not depressed.<br><br>Correlational analysis using Spearman's Rho for categorical variables showed strong positive associations between depression scores (yes/no) and persistence of concussion symptoms (yes/no) over 3 months.<br><br>There was no significant difference between depressed/non-

									depressed children who had experienced multiple concussions.<br><br>Correlational analysis between depression scores (CDI T score recorded by child) and the number of concussion symptoms reported were significant at visit one and three. Between CDIT-parent reported depression symptoms and the number of concussion symptoms reported were significant at all three visits.<br><br>Depression scores (CDI T score recorded by child) and experienced prolonged symptoms for 1 month were not significant for child or parent reported depressive symptoms and for experienced prolonged symptoms for 3 months was only significant for

									child but not parent report.<br><br>Logistic regression predicting likelihood of depression in concussion recovery with model including symptom score time-1, admitted to hospital, symptoms > 3 months, ≥2 concussions and gender was significant. The significant individual predictors in the model were high PCSI scores and admission to hospital.<br><br>Univariate analysis using length of hospital stay to predict depression found it to be significant.
O'Rourke 2016	70	US	mean age = 14.60, SD = 1.90	54% Female		Concussion	Anxiety	Longitudinal, no control group, non-interventional	Correlations:<br><br>At time point 1: Athletes report of performance anxiety, particularly concentration disruption and somatic anxiety and worry is not

									significantly correlated with symptom severity.<br><br>At time point 2: Athletes report of performance anxiety, particularly concentration disruption and somatic anxiety and worry is significantly correlated with symptom severity.<br><br>At time point 3: All subscales apart from worry were significantly correlated with symptom severity.<br><br>There was no significant correlation between concussion history or days since concussion and any of the anxiety scales.<br><br>Regression Concentration anxiety subscale was a significant predictors of Time 1 symptom intensity with higher reported concentration

									disruption predicting more intense symptom expression. The other subscales were not significant predictors.<br><br>Performance anxiety and the concentration and somatic anxiety subscales were significant predictors of Time 2 symptom intensity. Athletes who reported more performance anxiety, concentration disruption and somatic anxiety experienced more self-reported post-concussion symptoms at Time 2. Worry was not a significant predictor.<br><br>Performance anxiety, the concentration and somatic anxiety subscales were significant predictors of Time 3 symptom intensity. Worry was not a significant predictor.

									Performance anxiety and the subscales were not significant predictors of the time 1 to 2 change.<br><br>Performance anxiety and the concentration disruption and somatic anxiety subscales were significant predictors of Time 1 to 3 change. Greater anxiety predicted decreased symptom change and performance anxiety was a significant predictor of symptom intensity change over and above age, gender, number of days since the last concussion and self-reported concussion history.
Merritt et al. 2015	932	US	Baseline (N = 846) M 18.53 SD 1.06<br><br>Post-concussion (N=86) M	Final baseline sample: Female 24.7%<br><br>Post-concussi on	Football Baseline- B 30.3% PC 43%<br>Men's basketball- B 6.7% PC 11.6%<br>Men's ice hockey- B 9.8% PC 5.8%<br>Men's lacrosse - B 15.1% PC 15.1%<br>Men's soccer- B	An injury to the head resulting from a trauma or biomechanical force wherein brain function is disrupted as evidenced by any alteration in mental status and/or post-	Affective symptom cluster and sleep symptom cluster.	Longitudinal, no control group, non-interventional	Results showed that there were no significant increases from baseline to post-concussion on the sleep or affective symptom score.

			19.98 SD 1.44	sample Female 17.4%	11.2% PC 4.7% Women’s basketball - B 4.6% PC 3.5% Women’s lacrosse - B 8.3% PC 7.0% Women’s soccer- B 10.9% PC 7.0% Wrestling -B 2.2% PC 2.3% Other B 0.9% PC 0%	concussion signs or symptoms at the time of injury, posttraumatic amnesia lasting less than 24hours, and/or loss of consciousness lasting 30 minutes or less.			
Roiger et al. 2015	21	US	19.8 ± 1.4 years (aged 18–22 years)	5% Female	Men's basketball (n= 1), football (n= 6) and wrestling (n= 13) and women's basketball (n= 1).	(1) participants who sustained physician-diagnosed concussions and (2) participants who sustained an injury other than concussion as defined by the NCAA Injury Surveillance Program and which resulted in 1 or more days of time lost from their respective sports.	Depression	Longitudinal, with control group, non-interventional	No significant differences in baseline CES-D scores were evident among the concussed, injured/non-concussed, and healthy control groups. Depressive symptoms in the concussed participants scores peaked at 1 week post-concussion and then continued to decrease until the 3-month time point. Although we found significant differences between depressive symptoms scores at baseline and 1 week post-concussion, none of the other assessment points differed from baseline.

									Depressive symptoms scores in the injured/non-concussed participants peaked at 1 week postinjury and remained elevated over baseline at the 1-month time point before decreasing toward baseline at 3 months.
Covassin et al. 2014	126	US	Total: M = 22.69 ± 1.75 years, range (18 to 24 years)<br><br>Concussion group 22.54 ± 1.73<br><br>Orthopaedic Injuries 22.84 ± 1.77	26% Female	Participants were active in: football (55.6%) men's wrestling (14.3%,) softball (6.3%) women's soccer (6.3%) women's volleyball (6.3%) women's basketball (4.8%) field hockey (3.2%) baseball (1.6%) men's basketball (1.6%).	Concussion injuries group matched with orthopaedic injuries group.<br><br>Lower body injuries accounted for 43 (68.3%) and upper body injuries for 20 (31.7%) of the orthopaedic injuries. Athletes from institution A sustained 44 concussions and 53 orthopaedic injuries, and athletes from institution B sustained 19 concussions and 10 orthopaedic injuries.<br><br>Concussion was operationally defined as “a complex	Anxiety (state and trait)	Longitudinal, with control group, non-interventional	Findings show no differences for the State-Trait Anxiety Inventory between the concussed and orthopaedic-injury groups.

						pathophysiological process affecting the brain, induced by traumatic biomechanical forces.			
Mainwaring et al. 2010	51	Canada	21.2 years (SD = 2.94; Range 17.5–37.0)	58.8% Female<br><br>Concussed group: 25% Female<br>ACL injury group: 85.7% Female<br>Un-injured group: 71.4% Female	Athletes from nine varsity sports teams (basketball, field hockey, football, hockey, lacrosse, mountain biking, rugby, soccer and volleyball)	1) Concussed athletes (2) athletes with confirmed anterior cruciate ligament injuries.<br><br>Concussion was diagnosed based on the following criteria developed by the research team: (1) Observed or reported acceleration/deceleration of the head; (2) Any observable alteration in mental status; (3) Observable signs such as confusion, vacant stare, poor coordination, difficulty concentrating, poor balance; and/or (4) Any self-reported symptoms such as headache, loss of consciousness, nausea, balance problems or difficulty reading or concentrating.	Mood disturbance and Depression	Longitudinal, with control group, non-interventional	Findings suggest that response to athletic injury is not a result of pre-morbid emotional dysfunction.<br>Both groups reported significant increases in depression scores post-injury compared with the uninjured group.<br>Concussed athletes reported significant changes in overall emotional disturbance post-injury compared with the un-injured athletes, but not when compared to the athletes with ACL injuries.<br><br>Athletes with ACL injuries reported over seven times more depression 11 days post-injury than at baseline. In contrast, 4 days post-injury concussed athletes

									showed elevated depression scores three times greater than scores at baseline, but the elevations resolved 1 week after injury. No statistical differences in patterns of recovery between injured groups and the un-injured controls.
Appaneal et al. 2009	164	US	(M = 19.7, SD = 2.0) range (14 to 24)	34% Female	Participants competed across 9 varsity sports at two universities. Sports in which participants were involved included football (46%), basketball (17%), soccer (15%), volleyball (9%), baseball (6%), gymnastics (4%), track and field (3%), and wrestling (1%).	We defined injury as physical trauma that resulted in restricted (no) participation for a minimum of one week. Types of injuries involved joints (e.g., ligaments and dislocations), muscle, bone (e.g., fractures), head (e.g., concussion), neck (i.e., cervical spine), and/or low back (i.e., disc). The most common injury sustained by participants involved the knee, including bone bruises or tears to the medial collateral and/or anterior cruciate ligaments (MCL, ACL).	Depression and diagnostic criteria for a mood and/or anxiety disorder.	Longitudinal, with control group, non-interventional	A self-report checklist and a clinical interview was used to compare depression among male and female athletes at 1 week, 1 month, and 3 months postinjury. Results revealed significant effects of group (injured vs. control) and time (since injury), and these effects were different for the two depression measures. Both athlete- and clinician-rated depression symptoms decreased over time. Clinician-based depression ratings for athletes with injuries

									exceeded those of healthy athletes at 1 week and remained elevated above healthy controls up to 1 month postinjury.
Steffen et al. 2009	157	Norway	15.4 years (SD=0.8, range 13–17)	100% female	Football	An injury was registered if it caused the player unable to fully take part in match or training sessions the day following the injury (time loss injury). Acute injuries were defined as injuries with a sudden onset associated with a known trauma, whereas overuse injuries were those with a gradual onset without any known trauma. A previous injury was defined as an injury of the same type and the same site as an index injury and that occurred after a player had returned to full participation from the index injury.<br><br>Per limb, the average number of previous injuries to the ankle, knee, hamstring and groin was 1.8 (2.7; 0–	life stressors, anxiety and coping strategies	Longitudinal, with control group, non-interventional	There were significant between-group differences to the disadvantage of previously injured players for use of emotion-focused coping strategies but not problem or behaviour focused strategies.<br><br>Players with an injury history perceived their anxiety reactions to be more debilitating for their performance than did uninjured players, but no significant difference for somatic reactions and concentration reactions.<br><br>Players with an injury history compared to uninjured players

						16).<br><br>A total of 296 of the 1430 players (20.7%) sustained at least one injury during the 2005 season. Of these players, 49 (3.4%), 16 (1.1%) and one (0.07%) incurred two, three and four injuries, respectively, leading to a total of 380 injuries. There were 330 acute injuries and 50 overuse injuries.<br><br>Head & Neck injury<br>Contusion 5<br>Sprain 1<br>Other 8<br>Total (n= 14) 4.2%<br><br>Upper body injury<br>Contusion 9<br>Sprain 5<br>Strain 4<br>Dislocation 1<br>Fracture 6<br>Other 2<br>Total (n= 27) 8.2%<br><br>Lower body<br>Contusion 64<br>Sprain 135<br>Strain 73<br>Dislocation 1<br>Fracture 1			experienced more stressful life events but not reaction to stressful life events.<br><br>Logistic regression models to predict new injuries found that the risk of an injury was increased for players with a high level of perceived life stress compared with those players with a presumed low level of life stress.<br><br>Coping strategies and anxiety subscales did not predict new injuries.

						Pain 1<br>Other 14<br>Total (n= 289) 87.6%<br><br>Part of lower body<br><br>Hip<br>Contusion 4<br>Total (n= 4) 1.2%<br><br>Groin<br>Strain 19<br>Total (n= 19) 5.8%<br><br>Thigh<br>Contusion 3<br>Strain 46<br>Total (n= 49) 14.8%<br><br>Knee<br>Contusion 19<br>Sprain 20<br>Strain 2<br>Dislocation 1<br>Other 11<br>Total (n= 53) 16.1%<br><br>Lower leg<br>Contusion 12<br>Strain 4<br>Other 1<br>Total (n= 17) 5.2%<br><br>Ankle<br>Contusion 13<br>Sprain 111<br>Other 1<br>Total (n= 125) 37.9%			

						Foot including toe<br>Contusion 13<br>Sprain 4<br>Strain 2<br>Fracture 1<br>pain 1<br>Other 1<br>Total (n= 22) 6.7%			
McGuine et al. 2019	125	US	mean ± SD age, 15.9 ± SD 1.1 years	36% female	All sports with special emphasis on basketball, football, ice hockey, soccer, volleyball, and wrestling.	A Sports-related concussion was defined as “trauma induced alteration in mental status that may or may not involve loss of consciousness.	Health related quality of life (HRQoL)	Longitudinal, no control group, non-interventional	High school athletes who sustained an SRC reported lower HRQoL immediately after the onset of their SRC. However, by return to play and through the immediate 12 months after their SRC, the majority of the athletes had HRQoL rates the same as or better than baseline levels.
Kontos et al. 2012	3189 3	US	The baseline sample (mean ± standard deviation) was 15.74 ± 1.78 years, (Range of 13 to 22 years), and the post-concussion sample was 17.14 ± 2.25 years, Range(13 to 24 years).	Total 46% Female<br><br>Baseline Female 47%<br>Post-concussi on Female 33%	sports with >1% representation are:<br><br>Baseline<br>American football (24.4%)<br>Soccer (19.5%)<br>Basketball (9.6%)<br>Lacrosse (9.1%)<br>Volleyball (5.9 %)<br>Softball (5.2%)<br>Track/cross-country (4.4%)<br>Cheerleading (3.9%)<br>Baseball (3.8%)	Sports related concussion	Headache, difficulty concentrating ,fati gue, drowsiness, mentally foggy, Feeling slowed down, Dizziness, Trouble falling asleep, Difficulty remembering, Irritability Sleeping more than usual, Sleeping less than	Longitudinal, no control group, non-interventional	Exploratory factor analytic (EFA) methods were applied to 2 separate samples of athletes who completed the PCSS at baseline and 1 to 7 days after a sport-related concussion. A 4-factor solution that included cognitive-fatigue-migraine, affective, somatic, and sleep was revealed for the

					Field hockey (3.2%)<br>Wrestling (2.6%)<br>Ice hockey (2.2%)<br>Tennis (1.5%)<br>Swimming/diving (1.2%)<br>Other (3.7%)<br><br>Post-concussion<br>American football (42.4%)<br>Soccer (18.3%)<br>Basketball (6.4%)<br>Wrestling (4.6%)<br>Lacrosse (4.4%)<br>Ice hockey (4.0%)<br>Softball (2.7%)<br>Field hockey (2.6%)<br>Baseball (2.4%)<br>Volleyball (1.7%)<br>Other (10.5%)		usual, Feeling more emotional, Nervousness, Sadness, numbness		post-concussion EFA. High school athletes reported higher baseline levels of the cognitive-sensory and vestibular-somatic symptom factors and lower levels of the sleep-arousal factor than college athletes.
Kontos et al. 2012	75	US	54 high school (mean age = 15.74SD = 1.28y) and 21 college athletes (mean age = 19.68 SD=1.33y).	32% Female		Sport-related concussion<br><br>Operational definition of concussion. Concussion was operationally defined as "a complex pathophysiological process affecting the brain, induced by traumatic biomechanical forces" and was required to meet the after criteria: (1) presence of on-field	Depression	Longitudinal, no control group, non-interventional	Concussed athletes exhibited significantly higher levels of depression from baseline at 2 days, 7 days, and 14 days post-concussion. Collegiate athletes demonstrated a significant increase in depression at 14 days post-concussion than did high school athletes.

						signs (e.g., posttraumatic amnesia and loss of consciousness) and symptoms (e.g., dizziness and headache) as determined by a sports-medicine professional trained to identify concussions, (2) decrease from baseline levels in at least 1 post-concussion neurocognitive score determined by reliable change estimates, and (3) increase from baseline levels in post-concussion symptoms determined by reliable change estimates.			
Le et al. 2021	253	Canada	median age was 23 years (range = 14–29)	55% Female	Soccer was the most common pre-injury sport (35%) with ice hockey (21%), basketball (12%), skiing or snowboarding (8%), football (5%), rugby (4%), running (4%), volleyball (4%), dance or gymnastics (2%), horseback	Sport-related knee injury (clinical diagnosis of a ligament, meniscus, or other intra-articular tibiofemoral or patellofemoral injury)<br><br>Type of injury<br>56% ACL tear all of whom underwent	Health related quality of life (mobility, self-care, usual activities, pain/discomfort, and anxiety/depression) as well as health scale and knee-specific HRQoL.	Cross-sectional, with control group, non-interventional	Our findings indicate that a 3–12 year history of a youth sport-related knee injury is not associated with generic HRQoL but is negatively associated with condition-specific HRQoL. A previous ACL tear was also

					riding or rodeo (2%), baseball (1%), figure skating (1%), lacrosse (1%), and field hockey (1%) also identified.<br><br>Main sport<br>Uninjured 35% 95%CI (27 - 44)<br>Injured 35% 95%CI (27 - 44)	ACL reconstruction. (16%) had meniscus injuries,<br>(12%) had other ligament injuries (i.e., grade I-II ACL or posterior cruciate ligament injury, grade I-III medial or lateral collateral ligament injury)<br>15% had a patellofemoral subluxation or dislocation<br>(2%) had a fracture.			associated with a poorer condition-specific HRQoL
Martin et al. 2020	637	US	Median age = 13 years (IQR 11-15) Range 5 to 17 years	53% female		mild traumatic brain injury (mTBI) defined<br>as a provider-diagnosed concussion based on clinical presentation in accordance with previously described criteria: mild, non-penetrating brain injury directly or indirectly caused by a biomechanical force, with associated transient neurological or functional disturbance. The terms mTBI and concussion are used interchangeably.	Anxiety disorder	Longitudinal, with control group, non-interventional	Pre existing anxiety disorders are associated with higher mean concussion and vision symptom scores both immediately following injury and throughout the recovery period. Pre-existing anxiety disorders are a risk factor for prolonged symptom recovery as well as extended time to return to school and physical activity without accommodations, in univariate analysis and after controlling

									for age, sex and time.
Coffman et al. 2021	55	US	Mean 14.5, SD 1.4 years range (12-17 years)	43.6% Female		Concussion	Emotional clinical symptoms (emotional (e.g., irritability, frustration, and restlessness) and depressive symptoms and Neurobehavioral function (Inhibit, Shift, and emotional control).	Longitudinal, no control group, non-interventional	Clinical (emotional) and depressive symptoms significantly decreased from the subacute to post-acute evaluation. There was no significant difference for the Behavioural Regulation Index from the subacute to post-acute evaluation.
Garcia et al. 2021	306	US	Mean 15.7 SD 1.1 Range 10-19 years.	Female 57.5% Unspecified 7.5%	Inclusion criteria included long-distance running activities including team/club cross country, track and field (distances ≥800 m), road races, or recreational running.<br><br>Participants primary sports included:<br>Cross-Country<br>Track<br>Unspecified<br>Soccer<br>Basketball<br>Swimming<br>Baseball/Softball<br>Lacrosse<br>Dance<br>Nordic Skiing	Running related injury.<br>A RRI was defined as pain in the low back or lower limbs that caused either a restriction/stoppage of running (distance, speed, duration, or training) for at least 7 days or 3 consecutive scheduled sessions, or required the participant to consult a physician or other health professional<br><br>lower leg 23.8%<br>knee 23.4%<br>ankle/foot 21.6%<br>upper leg 19.0%<br>low back 12.1%	Health related quality of life and sleep quality.	cross-sectional, no control group, non-interventional	No significant differences in frequencies long-distance runners reporting quality of life problems among injury status were found. No significant differences of sleep duration or quality among injury status. Middle- and high-school runners with a current or previous injury reported significantly lower overall health than uninjured runners.

					Tennis<br>Boxing<br>CrossFit<br>Field hockey<br>Gymnastics<br>Martial Arts<br>Wrestling				
Vassilyadi et al. 2015	35	Canada	Age 10-17.9 median 15.4	Female 40%	Hockey 43%<br>Other Sport 21%<br>Rugby 8%<br>Football 8%<br>Gym Class/Training 6%<br>Basketball 6%<br>Tobogganing 6%	Concussion - sports related.	Quality of life	Cross-sectional, no control group, non-interventional	No significant difference were found in mean PedsQL score between children who had less than 2 head injuries compared with children who had 3 or more head injuries and bivariate correlations between mean symptom severity scores and the PedsQL total score found the following symptoms negatively correlated with the PedsQL total score at a level of significance ringing in the ears, trouble falling asleep, memory problems, poor concentration, depression, poor balance, sadness, headache, nausea, vacant stare/glassy eyes, dizziness and feeling in a fog.

Clacy et al. 2020	259	Australia	M = 13.0, SD = 1.8, range 11–17 years	100% Male	Rugby.<br><br>The majority of the participants (78%) also participated in other forms of sport, namely other codes of football (e.g., rugby league, Australian Football League, soccer; 27%), or another team ball sport (e.g., basketball, volleyball, cricket; 17%).	Sport-related concussion (SRC)<br><br>59% were diagnosed by team medical aides ("medics"); 25% were diagnosed by hospital/emergency room doctors, and 15% were diagnosed by family doctors/general practitioners).	ADHD, socio-cognitive development, emotional functioning, Attentional, motor, and non-planning impulsiveness and emotional, behavioural, and metacognitive skills	Cross-sectional, no control group, non-interventional	There was no statistically significant difference in psychological factors between participants with a history of SRC and those without
Valovich et al. 2009	205	US	uninjured (age = 16.0 ± 1.1 years)<br><br>injured (age = 15.9 ± 1.1 years)	Uninjured - 52% Female Injured - 55.5% Female	Baseball, Basketball, Cheerleading, Football, Golf, Soccer, Swimming/Diving, Tennis, Track/Cross-country, Volleyball, Wrestling.<br><br>Thirty-one percent of our injured group and 31.3% of our uninjured group indicated that they participated in multiple interscholastic sports.	65.5% of injuries were to the lower extremity, followed by the upper extremity (29.1%) and head or spine (5.5%).	Quality of life mental health subscale and happiness.	Cross-sectional, with control group, non-interventional	On the SF-36, the injured group demonstrated lower scores for physical functioning, limitations due to physical health problems, bodily pain, social functioning, and the physical composite. On the PODCI, the injured group reported lower scores on the pain and comfort subscale and the global score. No significant differences between groups for mental health subscales and happiness subscale.

Cuff et al. 2022	4937	US	range 10–18 years<br><br>10 - 4.6%<br>11 - 8.1%<br>12 - 10.6%<br>13 - 14.1%<br>14 - 17.2%<br>15 - 17.3%<br>16 - 14.5%<br>17 - 10.4%<br>18 - 3.3%	39% Female		Concussions<br><br>Defined as a bump, blow or jolt to the head and neck region or other area of the body, caused by biomechanical forces, resulting in a transient disturbance to the brain.	Prior psychiatric diagnoses (i.e., ADD/ADHD, MDD, learning disorders, anxiety disorders).	Longitudinal, no control group, non-interventional	Psychiatric comorbidities prior to injury were associated with higher risk of prolonged recovery from concussion. ADD/ADHD was associated with higher risk of prolonged recovery (>28 days) and prior diagnosis of anxiety was associated with greater risk of extended recovery (>90 days).
Marshall et al. 2019	759	Canada	Mean age was 15.5 years old (range 13–25).	40.7% females	Hockey 44.7%<br>Soccer 9.9%<br>Football 10.8%<br>Basketball 5.5%<br>Volleyball 2.6%<br>Baseball 0.7%<br>Motor Vehicle Accident 0.7%<br>Winter sports 3.8%<br>Martial arts and wrestling 2.4%<br>Rugby 6.1%<br>Falls 2.2%<br>Lacrosse 1.4%<br>Cheerleading 1.8%<br>Assault 0.3%<br>Other sport 5.4%<br>Other 1.7%	Concussion	Previous history of depression and anxiety	Longitudinal, no control group, non-interventional	Relationship between failure of the test and self-reported history of pre-morbid anxiety were found to be statistically significant on initial presentation.<br><br>Pre-morbid anxiety was found to significantly predict the length of time between injury and RTP clearance.
Chrisman et al. 2019	30	US	Mean age 15.5 years (SD 1.6)	56.7% Female	Mechanism of injury. Football (16.7%), soccer (1.6.7%), basketball (13.3%),	Sport-related concussions (SRC) Consistent with Zurich definition by a	Health related quality of life and fear of pain and fear of avoidance.	Longitudinal, with control group, interventional	Health-related quality of life improved significantly for all

			range 12-18 years.		wrestling (10%), swimming (6.7%), volleyball (6.7%), softball (6.7%), lacrosse (6.7%), tennis (3.3%), hockey (3.3%), ultimate frisbee (3.3%), gymnastics (3.3%), dance (3.3%).	clinician experienced with concussion management.			participants and fear-avoidance of pain declined significantly for all participants, but neither was significantly different by intervention group.
Wilmoth et al. 2022	393	US	mean age 14.7 SD (1.6), range 12-18 years.	45% Female	Football (28%) soccer (23.7%) basketball (12.7%) Other (35.6%)	Concussion.<br><br>Clinicians adhered to international concussion consensus guidelines in determining concussion diagnosis	Anxiety, depression and sleep quality.	Longitudinal, no control group, non-interventional	Using Poisson regression greater anxiety symptoms, and greater depression symptoms conferred risk of longer recovery. Modelling recovery using survival analysis, which censors individuals for whom exact duration is unknown, yielded sex-specific findings such that depressive symptom severity was a predictor of recovery for male athletes. It is worth noting that anxiety and depressive symptoms emerged as predictors despite the relatively low mean scores. Premorbid psychiatric history

									did not predict protracted recovery.
Ali et al. 2021	7453	US	Mean 15.4 years, range(12–22) years	Female D/A only = 67.2% DA meds = 54.9% Non-D/A = 33.2%	Approximately 30% of both DA cohorts participated in football, whereas 40% of the non-DA cohort played football. Other sports included soccer (12% overall prevalence), basketball (9%), volleyball (6%), lacrosse (6%), baseball or softball (6%), cheerleading (4%), and wrestling (4%).	Concussion. Concussions were defined as blunt trauma to the head or face causing a rapid alteration of the individual's mental status and/or the appearance of multiple symptoms not present before the injury, such as headaches, dizziness, nausea, vomiting, and blurred vision.	Premorbid depression or anxiety (DA)	Longitudinal, with control group, non-interventional	Premorbid DA itself does not appear to affect concussion incidence, nor does it seem to affect the recovery or persistence of symptomatic and neurocognitive dysfunction Post injury. However, premorbid DA along with antidepressant use is associated with increased concussion incidence as well as elevated symptom scores and verbal memory scores up to 7 days after concussion.
Alsalaheen et al. 2022	981	US	range 13-18 median 15.6	45% Female		Concussion. Concussion diagnosis was determined by a physician using the most recent diagnostic criteria described in the Berlin Consensus Statement on Concussion in Sport.	Mental health subscale of the SCAT5 symptoms scale (more emotional, Sadness, Nervous or anxious, Irritability and Trouble falling asleep).	Cross-sectional, no control group, non-interventional	There is a five factor solution to the SCAT-5 scale, and this was consistent across sexes.
Lam et al. 2019	183	US	Positive knee injury group - age mean 15.7 SD	Control group (n = 147) Female 40.1%		Knee injury	Lower health-related quality of life (HRQOL)	Cross-sectional, with control group, non-interventional	Findings suggest that adolescent athletes with a previous knee injury experience lower

			[1.4] No knee injury history age mean 15.5 SD [1.4]	Knee injury group (n = 36) Female 34.3%					health related quality than their peers with no knee injuries. Specifically these individuals report more impairments and deficits in physical, school, and social functioning. However no significant differences were reported for the emotional functioning subscale.
Harriss et al. 2020	124	Canada	Sixty-nine concussed age 15.2, SD 1.6<br><br>55 control age 14.4, SD 1.7	42% female		Sport concussion	PCSS mental health symptom domains e.g., More emotional, Sadness, Nervous or anxious, Irritability and Trouble falling asleep.	cross-sectional, with control group, non-interventional	Collectively, PCSS scores could differentiate between concussed and non-concussed adolescents; however, findings indicated that there were no statistically significant differences in the proportion of individuals presenting with a concussion and controls for symptoms of trouble falling asleep, more emotional, irritability, sadness, and anxious. There were significant

									differences for headache, Head pressure, Fatigue, “Don't feel right", “In a fog", “Slowed down”, Difficulty concentrating, Nausea, Drowsiness, Dizziness, Confusion.
Bailey et al. 2019	15	US	Mean 15.75 years SD 1.39, age range 14-18 years	44% Female		Concussion	Depression	Longitudinal, with control group, interventional	Total depression endorsed was elevated across the sample at baseline and a significant difference in endorsed depression was observed between groups at baseline, with substantially higher depression at baseline for the intervention group than that for the control group.
Watson et al. 2021	2073	US	Mean 15.6 ± 1.1 years, range 14-18 years old.	100% Female	Volleyball	Injury location<br>Ankle 30%<br>Knee 14%<br>Shoulder 11%<br>Hand/fingers 10%<br>Head 7.4%<br>Upper leg/thigh 4.9%<br>Foot/toes 4.4%<br>Lower back 3.9%<br>Hip 3.4%<br>Wrist 2.5%<br>Lower leg 2.0%<br>Upper back 2.0%	Quality of life and sleep duration	Longitudinal, no control group, non-interventional	During the season, injured athletes demonstrated a greater decrease in total QOL compared with uninjured athletes. Athletes who sustained a season-ending injury had a significantly greater decrease in total QOL compared with injured athletes

						Elbow 1.0%<br>Pelvis 1.0%<br>Abdomen 0.5%<br>Chest/ribs 0.5%<br>Face 0.5%<br>Neck 0.5%<br><br>Injury type<br>Ligament sprain 42%<br>Muscle/tendon strain 20%<br>Tendonitis/tenosynovitis 17%<br>Concussion 7.4%<br>Contusion 4.4%<br>Fracture—acute 1.5%<br>Meniscus tear 1.5%<br>Subluxation 1.5%<br>Other 1.0%<br>Abrasion 0.5%<br>Dislocation 0.5%<br>Fracture—stress 0.5%<br>Labral tear 0.5%<br>Laceration 0.5%<br>Other 0.5%			who were able to return to play during the season. No significant interaction was identified between season-ending injuries and sleep duration, however .<br><br>Baseline sleep duration was an independent predictor of in-season injury among athletes. While the difference in sleep between those who went on to suffer an injury or not was relatively small, the relationship between sleep and injury persisted after adjustment for preseason total QOL score and grade.
Manuel et al. 2002	48	US	age 15-18 Although most of the participants were 17 years old (38%), age was relatively evenly distributed between 15	58% female	Male injured in:<br>football (56%)<br>baseball (11%)<br>wrestling (11%)<br><br>females injured in:<br>soccer (25%)<br>basketball (21%)<br>track (14%)<br>volleyball (7%)	The most common injury was to the knee (anterior cruciate ligament).	Depression, positive and negative stress and coping.	Longitudinal, no control group, non-interventional	There was a significant decrease in<br>depression scores over time, with significant decreases occurring from injury onset to 6 weeks, and from onset to 12 weeks. Injury severity significantly

			and 18 years.						predicted baseline depression in Injured Adolescent Athletes.
McLeod et al. 2022	122	US	Mean age 15.8, SD ± 1.1 years.	16.4% Female		Sport-related concussion.<br><br>Diagnosed per state's concussion legislation and based on the American Academy of Neurology definition.	Health-related quality of life (HRQOL) and fatigue	Longitudinal, no control group, non-interventional	Findings suggest that length of recovery is associated with the impact of concussion on perceptions of HRQOL among adolescents. Patients who sustained a concussion and had a prolonged recovery of greater than 13 days noted lower generic HRQOL, particularly in physical functioning, school functioning, and general, cognitive, and sleep fatigue. Furthermore, findings suggest that the impact of concussion on HRQOL is minimal and transient in patients who recover within a week. While length of recovery is associated with HRQOL in the first 2 weeks post-concussion, generic

									and specific HRQOL recovered in all groups by 30 days following the injury, which suggests that, in the long-term, most patients improve on these measures and suffer few lasting effects.
Houston et al. 2016	122	US	Mean 15.8 ± 1.1 years	16.4% Female	The majority of the sample included football (63.9%) and soccer (9.0%) athletes. The remainder of the sample comprised athletes from nine other sports (27.1%).	Concussion (sport-related concussion)	Quality of life and fatigue	longitudinal, no control group, non-interventional	HRQoL and symptom severity are highly correlated within each measurement period. For concurrent regression analyses at Day 3, PedsQL-Physical accounted for 17.9% of the variance in time lost beyond that accounted for by traditional measures. At Day 10, PedsQL-School accounted for 15.2% and symptom severity for 7.1% of this variance. In predictive analyses, at Day 3, PedsQL-Physical accounted for 3.9% and MFS-General for 3.3% of the variance in time lost beyond that accounted for by

									traditional measures. At Day 10, MFS-Cognitive accounted for 12.0% of this variance.
Beauchamp et al. 2019	311	Australia	Age 6–18 years<br>Median 11.9<br>IQR (9.1, 14.2)	35% female	Mechanism of injury:<br>Sports/recreational injury in 63.7%<br>Non-sport/fall 32.8%<br>MVC 2.3%<br>Assault 1.3%	Concussion<br><br>Defined by Zurich consensus statement (McCrory et al., 2009).	Quality of life and past history of mood sleep or developmental problems and disorders.	Longitudinal, no control group, non-interventional	At both 4 and 12 weeks post-injury, the most common reason for being less well was a combination of PPCS and lower quality of life.<br><br>The findings of the study indicate that absence of pre-morbid cognitive and developmental problems (learning disabilities and ADHD) is associated with a greater probability of wellness after paediatric concussion.<br><br>The absence of mood and sleep disorders was on the borderline of significance as a predictor of being well.<br><br>Children who scored higher on the SAC concentration

									subtest were more likely to be well after concussion. This test of working memory assesses performance based on backward digit span and recitation of the months of the year in reverse order.<br><br>No components of the acute ED ratings of PCSI unambiguously emerged as being especially useful in predicting wellness. The PCSI physical factor score, which taps into symptoms such as dizziness, balance problems, sensory sensitivity, and nausea, was initially identified as a predictor based on the Wald chi-square statistic, but the relation was not in the expected direction (greater presence of physical symptoms predicted wellness).<br><br>The PCSI physical factor score (symptoms of

									impaired concentration, memory, and speed of processing) was no longer predictive when the adjusted odds ratios were considered. There was no strong evidence for an overall effect, given that the Wald chi-square statistic was not significant and thus the effect of this variable may only be modest at best.
Hilt et al. 2022	200	US	Mean 14.7 years SD (1.70) range (11 -18 years).	62% Female		Sports and recreational related concussion and at least 3 persistent post-concussive symptoms (PPCS) more than 1 month after injury.	Traumatic life events, History of anxiety or depression symptoms, pre-event psychotropic medications.	Longitudinal, with control group, interventional	Greater odds of high-persistent versus recovery group trajectory membership was observed for adolescents with a pre-injury history of anxiety and/or depressive disorders.
O'Rourke et al. 2017	51	US	Age = 14.53 years, SD = 1.85	52.9% Female	Participants played in the following sports: soccer 24%, lacrosse 10%, American football 8%, basketball 8%, with the remainder playing in various other sports such as skiing, volleyball, hockey, swimming,	Concussion	Sport-Anxiety (somatic anxiety, worry, concentration disruption, performance trait anxiety) and athletic identity	Longitudinal, no control group, non-interventional	Psychosocial factors were meaningfully related to changes in symptom reports over a period of at least 21 days, accounting for substantial variance even when previously (and currently) demonstrated

					Frisbee, cheerleading, and wrestling.				demographic and concussion history variables were statistically controlled.<br><br>Athletic identity, amotivation, and performance anxiety bore the strongest relations to changes in self-report symptoms associated with concussion recovery, all of them associated with more severe symptoms.
Chrisman et al. 2019	863	US	5-14 Years<br><br>Child age (y)<br>5-7<br>11.1%<br>8-10 34.8<br>11-12 36.0<br>13-14 18.1	1% Female	American Football	Concussion (Concussion was defined based on the Zurich concussion consensus documents)	mental health history (history of diagnosed depression, anxiety, and/or attention deficit disorder	Longitudinal, with control group, non-interventional	Youth with depression had a 5-fold increased risk of concussion. When adjusted for all covariates and without depression: concussed and non-concussed athletes did not differ regarding history of attention problems, headaches/migraines or anxiety.
Eagle et al. 2022	68	US	12–18 years<br><br>Concussed group 15.2 ± 1.8 years	Female (concussed group: 29.8%; control		Sport-related concussion (SRC)<br><br>Participants were diagnosed with an SRC if they presented	Medical history (yes/no; ADD/ADHD, anxiety, depression), depression	Cross-sectional, with control group, non-interventional	There was no difference between the concussed and control group for history of

			Non-concussed group 14.6 ± 1.9 years	group: 50.0%)		with a clear mechanism of injury and subsequent signs and/or symptoms of SRC not otherwise explained by existing comorbidities.	symptoms and anxiety symptoms.		ADHD/ADD and anxiety.<br><br>The concussed group had significantly higher depression scores compared to the non-concussed and there was no significant difference in anxiety scores between the concussed group and the non-concussed.
McKay et al. 2013	316	Canada	The median age of players was 15 years (range, 13-17 years).<br><br>Specifically - Bantam (age, 13-14 years), and Midget (age, 15-17 years)	100% Male	Ice hockey	Hockey Injury<br><br>In total, 143 injuries were reported. Ninety-seven players reported 1 injury, 17 players reported 2 injuries, and 4 players reported 3 injuries. The top 3 injury types were concussion (22.38%; 95% CI, 15.84-30.10), muscle strain (14.69%; 95% CI, 10.37-20.42), and joint/ligament sprain (14.69%; 95% CI, 10.37-20.42). Of these injuries, Bantam players reported 57 injuries (57/132 = 43.18%; 95% CI, 34.59-52.08), and Midget	Body checking behaviours, anxiety and fear of injury.	Longitudinal, with control group, non-interventional	Those with low levels of athletic identity scores were at higher risk of first injury than those with high levels of athletic identity scores.<br><br>Strong positive attitudes of body checking did not influence risk of injury in the present study.<br><br>State anxiety did not predict injury.<br><br>As the CSAI-2R mean score did not change between the baseline and postinjury

						players reported 61 injuries (61/184 = 33.15%; 95% CI, 26.40-40.46). The injury rate for Bantam was 3.64 injuries per 1000 hours (95% CI, 2.83-4.61), and for Midget it was 4.03 injuries per 1000 hours (95% CI, 3.17-5.05).			measurements for Bantam players, and the change for Midget players was small, incurring an injury did not seem to affect state anxiety levels.<br><br>Reinjury fear is not associated with subsequent injury.
Champigny et al. 2022	517	US	Baseline age<br><br>High Anxiety Group (n = 19) M (SD) 15.60 (1.33) years<br><br>Low Anxiety Group (n = 38) M (SD) 15.55 (1.34)<br><br>Age post injury<br>High Anxiety Group<br>M (SD) 15.82 (1.43) years<br><br>Low Anxiety Group<br>M (SD)	63% Female in both the high anxiety and low anxiety group.	Current sport, n (%)<br>High anxiety vs low anxiety group<br><br>Baseball/Softball<br>HA 5.3%, LA 2.6%<br><br>Basketball<br>HA 0%, LA 7.9%<br><br>Cheerleading<br>HA 5.3%, LA 5.3%<br><br>Field hockey<br>HA 10.5%, LA 10.5%<br><br>Football<br>HA 21.1%, LA 18.4%<br><br>Lacrosse<br>HA 0%, LA 2.6%<br><br>Skiing/snowboarding<br>HA 15.8%, LA 5.3%<br><br>Soccer<br>HA 26.3%, LA 28.9%	Concussion	Pre-existing anxiety symptoms	Longitudinal, with control group, non-interventional	The high anxiety group endorsed a greater number of symptoms than the low anxiety group and rated symptoms as more severe across testing times. Using a modified symptom score that excluded anxiety-like symptoms, a mixed analysis of variance indicated a group by injury interaction; the high anxiety group reported greater increases in overall symptom severity following injury.

			15.81 (1.34) years		Ice hockey<br>HA 5.3%, LA 5.3%<br><br>No response<br>HA 10.5%, LA 10.5%				
Williams et al. 2021	70	US	age = 15.7 [0.9] years<br>Age range 14-17 years.	Females 10%<br>Unreported 17%	Sports<br><br>Football (52.8%)<br>Basketball (4.3%)<br>Cheer (4.3%)<br>Soccer (1.4%)<br>Wrestling (2.9%)<br>Volleyball (2.9%)<br>Unreported (31.4%)	Sports related concussion.	Health related quality of life (anxiety, depression, fatigue and pain interference).	Longitudinal, no control group, non-interventional	Overall significant improvements were found for Physical function mobility, anxiety, depression, fatigue and pain intensity subscales between day 3 and day 10, but no significant difference was found for peer relationships.<br><br>Significant differences were also found all subscales between day 3 and return to play. Significant differences were found at day 10 compared to return to play on the physical function mobility, anxiety, depression, fatigue and peer relationships subscales, while no significant difference was found for pain intensity.

									In secondary analyses none of the Pediatric-25 subscale scores at the any of the 3 time measurements were statistically associated with concussion history.
Du Preez et al. 2017	682	Australia	Pre-season age Mean 21.3 SD (3.6)<br>In season age Mean 21.5 SD (4.0) years	100% Male	Rugby	Concussion	Depression and anxiety	Cross-sectional, no control group, non-interventional	Players with greater or equal to 3 self-reported concussions had over two times higher odds of positive depression scores than those with 2 or less concussions in the preseason sample. The in-season sample had a similar odds although this did not reach statistical significance.
Li et al. 1995	2333	US and Canada	Age<br><br>0-4 years (5.7 %)<br>5-9 years (46.5 %)<br>10-14 years (47.8 %)<br><br>Age range (0-14 years)	24% Female	Bicycle riding	Head injury<br><br>Defined as any injury listed in the first five diagnoses that was coded as skull fracture and/or intracranial injury (International Classification of Diseases, 9th rev., Clinical Modification	Pre-existing mental disorder (e.g., disorder of neurohypophysis or attention deficit)	Cross-sectional, no control group, non-interventional	With and without adjustment for age, sex and motor vehicle involvement, children who had pre-existing mental disorders had a significantly increased likelihood of sustaining head injuries.

						N codes 800, 801, 803, 804, and 850-854).<br><br>Bicycle-related injuries (i.e., injuries that occurred while riding a bicycle).<br><br>54% of patients experienced a head injury.<br><br>Fracture of vault of skull 8.3%<br>Without intracranial injury 5.5%<br>With intracranial injury 3.8%<br><br>Fracture of base of skull 11.5%<br>Without intracranial injury 8.3%<br>With intracranial injury 3.2%<br><br>Other/unspecified skull fracture 4.1%<br>Without intracranial injury 2.8%<br>With intracranial injury 1.3%<br><br>Concussion 48.2%<br>Without loss of consciousness 5.5%<br>With loss of consciousness 32.7%			

						Unspecified 10%<br><br>Cerebral laceration/contusion 4.7%<br><br>Intracranial haemorrhage 6.8%<br>Subarachnoid 1.4%<br>Subdural 2.0%<br>Extradural 2.5%<br>Other/unspecified 0.9%<br><br>Other/unspecified intracranial injury 16.4%<br><br>Head injury was the primary diagnosis for 44% of the study population. Other injuries that frequently occurred were neck fracture (13% of patients), fracture of humerus/radius/ulna (11%), fracture of face bones (10%), internal injury (10%), and femur fracture (9%). Multiple injuries were common: 27% had two, 18% had three, and 25% had four or more injuries. Among patients with head			

						injury, 83% also had other injuries, predominantly fractures to the limb and neck. Less than one-third (30%) of the study population sustained only one injury.			
Cengiz et al. 2021	260	Turkey	age range of 13-18 years	56.20%	Field Defence 41.2%<br>Ball-games 24.2%<br>Racket-games 7.7%<br>Shooting 10%<br>Others 16.9%	Place of injury<br>Lower extremity 56.5%<br>Upper extremity 43.5%<br><br>When injury occurred<br>During competition 17.6%<br>During training 82.4%<br><br>Phase in which injury occurred<br>Warm-up 16.5%<br>1st Half 28.8%<br>2nd Half 52.4%<br>Stretching 2.4%<br><br>Season in which injury occurred<br>Summer 33.5%<br>Winter 37.1%<br>Spring 19.4%<br>Autumn 10%<br><br>Use of equipment when injury occurred<br>Yes 27.1%<br>No 72.9%	quality of life	Cross-sectional, with control group, non-interventional	Participants who had no sports injuries have a significantly higher score of quality of life when compared to the ones with sports injuries.

Watson et al. 2018	75	US	Mean age (15.5 ± 1.6 years)	100% Female	Soccer	In accordance with the consensus statement on soccer injury registration definition of time-loss injuries, participants were asked to report any injury that occurred during training or a game that resulted in the athlete being unable to continue to participate.<br><br>Injury location<br>Ankle (39%)<br>Knee (19%)<br>Head (14%)<br>Upper leg (11%)<br>Foot (8%)<br>Back (6%)<br>Lower leg (3%)<br><br>Injury type<br>Sprain (61%)<br>Muscle strain (17%)<br>Concussion (12%)<br>Contusion (8%)<br>Fracture (3%)	Mood, fatigue, stress, soreness, sleep quality, sleep hours, TL.	Longitudinal, no control group, non-interventional	Mood was found to be an independent predictor of injury in youth female soccer players. In addition, decreased daily mood was an important predictor of in-season injury, even after controlling for the acute effects of training load. This study did not find any relationship between injury and sleep quality, quantity , stress and fatigue from the preceding night.
Owoeye et al. 2022	86	Canada	Median age 23 for injured range (17-30) and 24 for uninjured (range 17-30)	77% Female		Significant sport-related ankle sprain SAS<br><br>Consistent with the International Ankle Consortium's	Ankle-related quality of life	Cross-sectional, with control group, non-interventional	Previously injured participants demonstrated significantly poorer ankle-related quality of life compared to controls.

						position statement, we defined an SAS as a clinical diagnosis of an ankle ligament injury; specifically including injuries involving the lateral ligament, ligaments of the tibiofibular syndesmosis (high ankle sprain), and/or medial ligament sprain that resulted in disruption of regular youth (i.e., ≤18 years of age) sport participation.			Previously injured participants demonstrated significantly higher fear of pain compared to controls.<br><br>No significant differences were found between injury history and athletic identity.
Timpka et al. 2022	480	Finland	mean age of 18.7 years (range 17.1–19.8 years).	48% Female	Athletic athletes	Sports injuries. The most common injury cause was overuse (91.8%).	Depression	cross-sectional, no control group, non-interventional	Pathways to depression caseness and depression predisposition were observed for injury history.
Kercher 2022	91	US	Mean age 15.31 (SD 1.02) range (13.26 – 17.74)	100% Male	High school tackle football	Sub-concussive head impact exposure.	Psychological need satisfaction (autonomy, competence and relatedness), depressive symptoms, anxiety symptoms, and thriving (happiness, accomplishment and supportive and rewarding relationships).	Longitudinal, no control group, non-interventional	Season-long impact exposure and age of first exposure to tackle football were not associated with mental health or thriving outcomes at post-season or change scores from pre- to postseason.

Charpentier 2021	153	US	Aged 12 -18 years.	49% Female		Diagnosed with sports related concussion	Somatic symptoms, depression and anxiety, previous and current psychological health conditions.	Longitudinal, no control group, non-interventional	Correlation analysis found that previous Concussion was not correlated with anxiety or depression. While anxiety, pre-injury somatization and depression were significantly correlated with both concussion symptom burden (PCSS) and protracted recovery.<br><br>None of the mental health variables from the main hypotheses were found to be significantly associated with protracted recovery in the Main Effect Analyses.<br><br>In moderation analyses pre-injury Somatization and anxiety scores were independently associated with a significantly increased likelihood of protracted recovery.
Huysmans &	117	US	Mean 19.50 (SD = 1.57) years range	30.8% Female	Men's team was football and women's teams were	Sport Injury<br><br>Injuries were defined	Life stress, competitive trait anxiety, self	cross-sectional, no control group,	In correlation analyses frequency of injury was

Clement 2017			(18-26 years)		volleyball and soccer<br><br>Football players comprised the majority of the study participants (n = 81, 69.2%), followed by women's soccer (n = 23, 19.7%) and women's volleyball (n = 13, 11.1%).<br><br>Approximately 35.0% (n = 41) of the athletes were starters on their team, and 40.2% (n = 47) were second string.	as all types of injuries that occurred in connection with sport participation that resulted in at least 1 day of missed or modified (i.e., reduced participation, strapping, etc.) practice or competition (Andersen & Williams, 1999), and/or required treatment.<br><br>Type of injury:<br><br>Ankle/foot/Achilles 25%<br>Knee 11%<br>Upper leg 13%<br>Arm 8%<br>Hand 5%<br>Shoulder 18%<br>Back 3%<br>Concussion 5%<br>Other 3%<br><br>Upper leg = hamstring, quadriceps, groin; Arm = elbow, biceps, and triceps.	compassion, coping and coping style.	non-interventional	significantly associated with positive stress.<br><br>Frequency of injury not significantly associated with self-Compassion, negative stress, Emotion-focused coping, avoidance-focused coping, Problem-focused coping, worry, somatic anxiety, concentration disruption, Athletic Coping Skills Inventory (ACSI) total score.<br><br>Severity of injury was positively associated with problem-focused coping but was not significantly associated with self-compassion, life events negative stress, positive stress, emotion focused coping, avoidance-focused coping, worry, somatic anxiety, concentration disruption and athletic Coping

									Skills Inventory (ACSI) total score.<br><br>Multiple Linear regressions model of all variables were found to be nonsignificant in a regression equation for frequency of injury and severity of injury.<br><br>Positive life stress was the only significant predictor of frequency of injury, while self compassion, negative stress, emotion focused coping, avoidance focused coping, problem focused coping, somatic anxiety, worry and ACSI total score did not significantly predict frequency of injury.<br><br>There was no significant predictors of severity of injury (positive life stress self compassion, negative stress, positive stress,

									emotion focused coping, avoidance focused coping, problem-focused coping, SAS worry, SAS somatic anxiety, and ACSI total score did not significantly predict frequency of injury.
Vargas et al. 2015	126	US	Concussed 18.4 ± 0.8 Control 18.9 ± 0.9	31.8% Female	football 44% lacrosse 21% basketball 13% soccer 10% ice hockey 8% wrestling 4%	Sport related concussion	Post concussion depressive symptoms	Longitudinal, with control group, non-interventional	More athletes reported clinically important depression symptoms after concussion than at baseline. For the control group, depression symptoms were not significantly different. Baseline postconcussion symptoms and number of games missed due to concussion were found to be a predictor of postconcussion depressive symptoms (PCDS). During follow-up analysis, controlling for history of psychiatric treatment, postconcussion

									symptoms, remained a predictors of PCDS whereas number of games missed due to concussion did not. Number of previous concussions were not found to be predictors of postconcussive depressive symptoms. In the control group, a predictor of time 2 depression symptoms were the number of previous head injuries. To evaluate the change in depression within an individual over time, we conducted reliable change analyses and found that concussed athletes were more likely to show a reliable increase in depression symptoms than nonconcussed control participants.
Ivarsson et al. 2013	56	Sweden	Range between 16-36 years (mean 25.05, SD = 5.46) years.	32.1% Female	Soccer	Injury<br><br>A player was defined as injured defined if he or she missed at least one practice or	Somatic trait anxiety and psychic trait anxiety, life events, daily hassle and uplift	Longitudinal, no control group, non-interventional	1. A path analysis was conducted examining the influence of personality traits (i.e., trait

						competition due to injury.	and coping strategies.		anxiety), state-level stressors (i.e., negative-life-event stress and daily hassles), and coping on injury occurrence. Results of the path analysis indicated that trait anxiety, negative-life-event stress, and daily hassle were significant predictors of injury among professional soccer players, accounting for 24% of the variance.<br><br>2) Regarding predictor variables - results indicated that there was a significant relationship between daily hassle and injury frequency but no direct relationship between maladaptive coping, trait anxiety and negative life event stress with injury frequency.
Johnson & Ivarsson 2011	108	Sweden	Range between 17 and 19 years old.	21.3% Female	Soccer	Injuries were defined as all types of injuries that occur in connection with sport participation.	State and trait anxiety, somatic anxiety, worry and concentration disrupters, coping,	Longitudinal, no control group, non-interventional	Injured athletes have a higher level of somatic trait anxiety than the non-injured athletes. Somatic

							negative life event stress, positive life event stress and total life event stress. scales of personality.		trait anxiety and mistrust were found to be significant predictors of injury occurrence. Injured athletes have a higher level of negative life event stress than the non-injured athletes. No significant differences in positive or negative coping between the injured and non-injured groups, as well as the result from the linear regression analysis between the predictor's negative coping, positive coping and injury as the dependent variable were found. Negative life event stress, somatic trait anxiety, negative coping, mistrust and stress susceptibility could explain 23% of the total variance and significant predictors were negative life event stress, somatic trait anxiety, negative coping and mistrust.

									Stress sensitivity was nonsignficicant.
Noh et al. 2005	105	South Korea	Mean age of 20.46 years (SD = 5.50).	96% Female	Ballet dancers	Injury<br><br>An injury was defined as any medical problem resulting from dance participation that restricted subsequent practice and performance for at least one day beyond the day the injury occurred.	Stress, anxiety, social support, and coping skills.	Longitudinal, no control group, non-interventional	1) Correlations between psychosocial factors and injury found that (peaking under pressure, goal setting/mental preparation, freedom from worry, confidence and achievement motivation) were significantly correlated with frequency of injury.<br><br>Positive, negative, minor and major life stress, Positive, negative, minor and major dance stress and somatic & worry anxiety, social support, coping with adversity and concentration were not correlated with frequency of injury.<br><br>2) Correlations between (negative dance stress, negative life stress, freedom from worry, confidence and achieve-ment motivation) were

									significant with injury duration.<br><br>Positive, minor and major life stress, Positive, minor and major dance stress and somatic & worry anxiety, social support, coping with adversity, peaking under pressure, goal setting/mental preparation and concentration were not correlated with frequency of injury.<br><br>The regression analysis identified freedom from worry and confidence & achievement motivation as significant predictors, accounting for 21% of the variance in frequency of injury. Peaking under pressure and goal setting & mental preparation were not significant predictors.<br><br>Findings from the regression analyses identified freedom

									from worry and negative dance stress as significant predictors of the variance in duration of injury. Negative life stress and confidence/achievement motivation were not significant predictors for duration injury.
Mac Donald et al. 2019	46	US	Control group Mean (SD) 12.9 ± 1.7 Concussed group Mean (SD) 13.3 ± 1.6 Range 10-14 years.	Control group (54%) Female Concussed group (59%) Female	Control group Basketball (17%), football (8%), Gymnastics (4%), Martial arts (8%), Soccer (39%), Softball/baseball (4%), Track and field (4%), Volleyball (4%), Wrestling (4%), Unknown sport (8%)<br><br>Concussed group: Basketball (9%), football (9%), Lacrosse (4.5%), Martial arts (4.5%), Roller Derby (4.5%), Rowing (4.5%), Soccer (37%), Softball/baseball (9%), Swimming (4.5%), Volleyball (4.5%), Unknown sport (9%)	Symptomatic concussion<br><br>Defined as patients who had sustained a concussion during sports or recreational play that was diagnosed by a treating physician, whose symptoms had remained unresolved after a minimum of 4 weeks post-injury, and who had been seen in the Sports Medicine, Concussion, or Rehab Medicine specialty clinics at Seattle Children's Hospital.	Overall health behaviour impairment and quality of life mental health symptoms of depression and anxiety and overall sleep impairment.	Longitudinal, with control group, non-interventional	The only domain in which an improvement was observed in the concussion patients that was significantly greater than that of the controls was on the symptom score derived from concussion symptoms. Concussion patients did exhibit sustained impairment in domains of psychological health and health behaviour at 6-month follow-up, which was significantly elevated in comparison which controls. Concussion patients had significantly higher

									severity of symptoms of depression as well as greater frequency of health behaviour impairment noted on the health behaviour inventory at 1-month post-injury and this remained the case at 6-month evaluation. There<br>was no difference in sleep performance as evidenced by the findings on the ASWS, or the GAD-7, which measures symptoms of anxiety. Overall measures of quality of life were also not distinguishing between groups at 6-month follow-up; however, this appears to be driven by the significant improvement in the concussion patients from initial evaluation, whereas the controls remained stable.<br><br>Initial concussion symptoms from the ImPACT was found to be a predictor of

									6-month overall quality of life across the 10 models consistently.<br><br>Initial concussion symptoms from the ImPACT and concussion diagnosis were also found to be consistent predictors across the 10 models of 6-month depressive symptom severity.
Legarreta et al. 2018	154	US	Mean age (SD)<br><br>FPH/PPH group (n=18) 15.28 SD (1.02) years FPH only (n=59) 14.92 SD (1.04) years Control (n=77) 14.73 SD (1.31) years	Female %<br><br>FPH/PPH group (66.7%) FPH only (35.6%) Control (45.5%)		Post-concussion symptom resolution (PCS) or persistence at 6 weeks.<br><br>PCS was defined based on the ICD-10 definition, which includes the presence of 3 or more symptoms without a defined time period.	ADHD/LD and psychiatric history (Anxiety, Bipolar disorder, Depression).	Longitudinal, with control group, non-interventional	Athletes with FPH/PPH compared with controls had an increased risk of PCS. Athletes with FPH only compared with controls also had an increased risk of PCS. Comparing athletes with FPH/PPH to athletes with FPH only, no added PCS risk was noted. Among various FPH diagnoses, anxiety and bipolar disorder were significantly associated with the presence of PCS.
Padaki et al. 2018	24	US	Mean 14.5 ± SD 2.7	50% Female	29.2% single-sport athletes, 58.3% of patients played	Anterior Cruciate Ligament Rupture (ACL injury)	Posttraumatic stress disorder	Cross-sectional, no control group,	Athletes experienced high levels of PTSD symptoms

			years, ≤21 years of age		multiple sports with a favourite; and 12.5% balanced their sports equally.		(PTSD) symptoms	non-interventional	which the author describes as being in contrast to normative values that other authors have established. Patients with athletic identity scores >50 incurred more psychological trauma, but this finding was not statistically significant.
Meier et al. 2016	76	US	Concussed athletes mean 21.0 SD (1.5) years<br><br>Non concussed athletes mean 20.2 SD (1.2) years<br><br>Healthy controls mean 21.9 SD (2.2) years	100% Male	Football athletes and one track-and-field athlete with extensive football experience (nine years), including experience at the collegiate level<br><br>Other sports healthy controls<br>Non-athlete 20<br>Cross Country/track 6<br>Basketball 1	Concussion history<br><br>(defined as concussions that were medically diagnosed by a clinician at the time of injury)	Self-reported happiness, anger, anxiety, restlessness, vigour and fatigue.	Cross-sectional, with control group, non-interventional	Healthy controls reported significantly higher happiness levels than both football groups (self-reported concussion history and no self-reported concussion history). There was no significant differences between the three groups on self-reported depression, anger, anxiety, restlessness, vigour or fatigue mood scores.
Tiric-Campara et al. 2012	55	Bosnia and Herzego vina	Mean age of 20.2 +/- 3.8 years. (13-28 years).	18.2% Female	kick boxers, karate fighters, and boxers (4 participants were coaches and 51 participants were fighters) .	Sports injury	Aggression and anxiety.	cross-sectional, no control group, non-interventional	There was a positive correlation between verbal manifested, physical latent subscales as well as total physical aggression score

									with the number of injuries of the respondents.<br><br>There was no positive correlation between physical manifested, indirect shift and verbal latent aggression subscales with the number of injuries of the respondents.<br><br>There was a significant positive correlation between levels of anxiety with the number of injuries of respondents.
Keightley et al. 2014	30	Canada	Concussed (mean age, 14.47±2.29 years)<br>Healthy controls (mean age, 14±2.3 years)	Concussed participants 53.3% Female<br><br>Healthy controls 46.7% Female<br><br>Overall 50% Female	Gym, soccer, football, ringette, swimming, trampoline and skiing.	Concussion<br><br>Mechanism of injury (Hit wall in gym class, kicked in head playing soccer, fell playing soccer, Fell playing ringette, Hit head while swimming, Hit head playing football, Hit head against wall while swimming, Hit in head by ball in soccer, Fell playing ringette, Struck on head by a knee while	Participants' self-perceived levels of anxiety and depression. Parent reported behaviour and emotional functioning domains (i.e., oppositional behaviour, inattention, hyperactivity) as well as competencies, adaptive functioning, and problems in child	cross-sectional, with control group, non-interventional	No significant differences were found between concussed participants and the control group on any of the ADHD features & problem behaviours, the Child Behaviour Checklist domains and participants' self-perceived levels of anxiety and depression.

						on trampoline, Hit in head by ball playing soccer, fell while skiing, Hit head playing football, Hit head playing soccer and Hit head playing football).	behaviour that map onto the major mood and behavioural disorders.		
Hammer et al. 2021	378	US	Mean age male 16.2<br>Mean age female 16.3<br><br>Total 16.3 SD (1.2)	36% Female	Male n= 80<br>Football n= 77.5%<br>Basketball n= 7.5%<br>Ice Hockey n= 5%<br>Soccer n= 3.75%<br>Wrestling n= 3.75%<br>Baseball n= 2.5%<br><br>Female n= 45<br>Volleyball n= 31.1%<br>Basketball n= 24.4%<br>Soccer n= 20%<br>Lacrosse n= 9%<br>Cheerleading n= 7%<br>Softball n= 4.5%<br>Field Hockey n= 2%<br>Ice Hockey n= 2%	Sport-related Concussion	Depressive symptoms	Longitudinal, no control group, non-interventional	Sport-related concussion did not worsen longitudinal measures of depressed mood in this cohort of high school athletes.
Ellis et al. 2017	399	Canada	Mean age 14.3 SD 2.3 years	40.3% Female	Hockey (n = 171) and soccer (n = 58) were the most commonly played sports at the time of injury.	Sports-related concussion<br><br>Defined according to the International Consensus on Concussion in Sport as an injury caused by transmission of biomechanical forces to the brain leading to clinical symptoms affecting multiple domains of physical,	History of depression.	longitudinal, no control group, non-interventional	Univariate analysis of clinical variables associated with the development of PCS among patients with acute SRC found no significance for past ADHD and history of learning disorder. However history of depression was found to be significantly associated with the

						cognitive, sleep, and neurobehavioral functioning.			development of PCS among patients with acute SRC.<br><br>On multivariate analysis, preinjury history of depression was found to be an independent predictor of postconcussion syndrome.<br><br>Previous diagnosis of ADHD, previous concussions, LOC, or previous diagnosis of learning disorders did not confound any of the observed associations.<br><br>Univariate analysis of clinical variables associated with the development of vestibulo-ocular dysfunction (VOD) among those with acute sports related concussion found no significance for History of ADHD and History of learning disorder but found significance for the history of depression.

									On multivariate analysis, significant predictors of vestibulo-ocular dysfunction (VOD) included preinjury history of depression.
DiSanti et al. 2022	85	US	(concussion: n = 21 male, n = 25 female; age = 15.1 ± 1.1 years;<br><br>Ankle sprain: n = 21 male, n = 18 female; age = 15.4 ± 1.3 years)	Concussion: 54.4% Female<br><br>Ankle sprain: 46.2% Female<br><br>Overall 50.6% Female	Football, Basketball, Volleyball, Cheerleading, Soccer, Baseball/softball Track, Lacrosse, Field Hockey, Gymnastics, Hockey, Other, Tennis, Wrestling	A concussion or an ankle sprain.<br><br>Ankle sprain group: Sprain/strain, ankle 41.2%<br>Tibiofibular ligament sprain 4.7%,<br><br>Concussion group: Concussion44.7%<br>Concussion with loss of consciousness 1.2%<br>Concussion, mental confusion without loss of consciousness 8.2%	Health Related Quality of life.	Longitudinal, with control group, non-interventional	Each injury group exhibited improved global and domain-specific PedsQL scores between their 2 measured time points (P < .05), indicating recovery.
Chau & Vilain 2021	1219	France	mean age =12.7 ± 1.3, range (9–18) years.	47.7% Female		School injuries (during the present school year)<br>School-physical/sports-training injuries 11%<br>Other-school-training injuries 4.9%<br>School-free-time injuries 7.6%<br>Out-of-school injuries (during the	Depressive symptoms	Cross-sectional, no control group, non-interventional	Depressive symptoms were strongly associated with one or more school-physical/sports-training injuries, other-school-training injuries, school-free-time injuries and out-of-school-sports injuries. The risk

						present school year)<br>Out-of-school-sports injuries 17.3%<br>Traffic injuries 2.3%			was higher for having two or more injury categories than for only one injury category. These results were robust and remained after further adjustment for socioeconomic features, obesity, alcohol use, tobacco use, and health status.
Abbott et al. 2019	25	UK	Mean ± SD: 20 years ± 1 year	100% Male	Soccer	Sports-related injuries of unclear types. An injury was defined as a period of being unable to complete at least 1 week of training.	Mental wellbeing	Longitudinal, with control group, non-interventional	The number of days missed to injury and not being selected for the games were the only variables found to be significant predictors of MW in the multivariate regression model. The length of time spent injured throughout the season had a significant negative effect on MW, and accounted for the biggest variance in MW (40%). When considered together, the length of time spent injured and not being selected to play games accounted for 50%

									of the variation in MW scores.
Leddy et al. 1994	343	US	M=20.42 years; SD=2.06; range 17 to 26 years	100% Male	Football, wrestling, baseball, track and field, gymnastics, cross-country, swimming, basketball, tennis, golf	injury was defined as physiological damage or body pain that required medical attention and caused an athlete to miss a game, practice session, or subsequent gameplay because athletic participation was impeded.<br><br>The most common injuries were to the knee, which accounted for 19.6% of all injuries, followed by the shoulder (16.9%), ankle (11.3%), leg (9.4%), arm (8.3%), hand (7.9%), back (6.0%), head/neck/face (5.7%), wrist (4.9%), pelvis/groin/hip (3.4%), chest/rib/abdomen (3.0%), foot (2.3%), and other (1.1%).<br><br>In regard to specific diagnoses, the ankle sprain was the most commonly diagnosed injury (9.9%),	Depression, state anxiety, physical self, and total self-esteem	Longitudinal, with control group, non-interventional	One-way MANOVAs found that the four injury groups were not significantly different in levels of depression, state anxiety, physical self, and total self-esteem prior to the beginning of the season.<br><br>Follow-up tests indicated that, following injury, the Injured and Recovered athletes displayed significantly higher levels of depression and state anxiety than the Noninjured and Late Injured athletes. At the same time, the Injured and Recovered athletes exhibited significantly lower total and physical self-esteem scores following injury than the Noninjured and Late Injured athletes.<br><br>Post-hoc tests

						followed by knee contusions (4.5%), fractured finger (4.2%), knee sprain (3.8%), back strain (3.4%), hamstring strain (3.4%), shoulder bursitis/tendinitis (3.0%), shoulder contusion (3.0%), elbow bursitis/tendinitis (3.0%), pelvis/groin/hip (3.0%), and knee cartilage damage (3.0%)			indicated that the Injured and Late Injured athletes had significantly higher levels of depression than either the Noninjured or Recovered athletes at the final evaluation. However, only the Injured group exhibited significantly higher levels of state anxiety at follow-up than the other three groups. Injured and Late Injured athletes exhibited significantly lower total and physical self-esteem scores than the Noninjured or Recovered athletes. In addition, the Late Injured athletes had significantly lower physical self-esteem scores than the other three groups.
Ford et al. 2000	121	Australia	Range: 16± 34 years. Mean ± s: 22.5 years ± 3.6years	46% Female		Sports-related injuries of unclear types. Injuries were defined as a medical problem sustained during practice or competition that	1) Life stress 2) Competitive trait anxiety 3) Hardiness	Longitudinal, no control group, non-interventional	Life stress was not associated with days missed through injury. Competitive trait anxiety was not correlated with sports injury.

						prevented participation (training or playing) for at least one day beyond the date of occurrence			Hardiness (composite) was significantly correlated with days missed through injury.
von Rosen & Heijne, 2019	386	Sweden	Range 15-19, median 17 years	47% Female	athletics, cross-country skiing, downhill skiing, freestyle skiing, handball, orienteering and ski-orienteering	Sports-related injuries of unclear types. Injury defined as any physical complaint that affected participation in normal training or competition, led to reduced training volume, experience of pain or reduced performance in sports	Subjective wellbeing	Longitudinal, no control group, non-interventional	Wellbeing and an injury self-report score were negatively correlated. Subjective well-being was significantly influenced by injury severity.<br><br>Subjective well-being the previous week predicted injury and injury severity the subsequent week, respectively. However substantial injury could not be predicted based on subjective well-being score.

Supplementary Material 4. Risk of Bias scores.

Study	Open Science	NHLBI	RoB	Robins
Psychological impact of sports injuries and psychological well-being in relation to sports performance in competition gymnasts.	No	Poor		
Presence and Perceptions of Menstrual Dysfunction and Associated Quality of Life Measures Among High School Female Athletes.	No	Fair		
Injured athletes' rehabilitation beliefs and subjective well-being: The contribution of hope and social support	No	Fair		
Self-reported injury in Australian young adults: demographic and lifestyle predictors.	No	Fair		

Exposure to Head Impacts and Cognitive and Behavioral Outcomes in Youth Tackle Football Players Across 4 Seasons.	No	Fair		
Quantitative Volumetric Imaging and Clinical Outcome Characterization of Symptomatic Concussion in 10- to 14-Year-Old Adolescent Athletes.	No	Poor		
A pilot study of active rehabilitation for adolescents who are slow to recover from sport-related concussion.	No			Serious
Effect of Collaborative Care on Persistent Postconcussive Symptoms in Adolescents: A Randomized Clinical Trial.	Yes		High	
Sports-Related Concussions and Adverse Health Behaviors Among Middle and High School Students	No			

Temporal Differences in Concussion Symptom Factors in Adolescents following Sports-Related Concussion	No	Fair		
Concussion Symptom Profiles among Child, Adolescent, and Young Adult Athletes	No	Poor		
Collaborative care for adolescents with persistent postconcussive symptoms: A randomized trial	Yes		Some concerns	
Initial somatic symptoms are associated with prolonged symptom duration following concussion in adolescents		Fair		
A Prospective Pilot Study of Anxiety Sensitivity and Adolescent Sports-Related Concussion.	No	Fair		

Individual Symptom Report Prevalence in Children and Adolescents With One, Two, and Three or More Persistent Symptoms After Concussion: A Brief Report.	No	Poor		
Psychosocial Outcomes of Sport Concussions in Youth Hockey Players.	No	Fair		
Mood, psychological, and behavioral factors of health-related quality of life throughout recovery from sport concussion.	No	Fair		
Clinical predictors of post-injury anxiety in adolescent patients following concussion.	No	Fair		
Depression in youth recovering from concussion: Correlates and predictors.	No	Fair		

Predicting symptom recovery post-concussion: An application of the integrated model of response to sport injury.	No	Fair		
A novel approach to classifying postconcussion symptoms: The application of a new framework to the Post-Concussion Symptom Scale.	No	Fair		
A longitudinal pilot study of depressive symptoms in concussed and injured/nonconcussed National Collegiate Athletic Association Division I student- athletes.	No	Fair		
Postinjury anxiety and social support among collegiate athletes: A comparison between orthopaedic injuries and concussions.	No	Fair		

Emotional response to sport concussion compared to ACL injury.	No	Good		
Measuring postinjury depression among male and female competitive athletes.	No	Fair		
Self-reported psychological characteristics as risk factors for injuries in female youth football.	No	Good		
The Effect of Sport-Related Concussion Injuries on Concussion Symptoms and Health-Related Quality of Life in Male and Female Adolescent Athletes: A Prospective Study	No	Fair		
A revised factor structure for the post-concussion symptom scale: Baseline and postconcussion factors	No	Fair		

Depression and neurocognitive performance after concussion among male and female high school and collegiate athletes	No	Poor		
What does the future hold? Health-related quality of life 3-12 years following a youth sport-related knee injury	No	Fair		
Concussion symptomology and recovery in children and adolescents with pre-existing anxiety	No	Good		
Predictive value of subacute heart rate variability for determining outcome following adolescent concussion	No	Fair		

Sport Specialization and Quality of Life among Middle- and High-School Long-Distance Runners of Different Injury Status: A Retrospective Study	No	Fair		
Symptom Experience and Quality of Life in Children after Sport-Related Head Injuries: A Cross-Sectional Study.	No	Fair		
Correlates of sport-related concussion in male junior rugby union: A concurrent analysis of biopsychosocial factors	No	Fair		
Recent injury and health-related quality of life in adolescent athletes	No	Poor		

Risk factors for prolonged recovery from concussion in young patients	No	Fair		
The use of an intensive physical exertion test as a final return to play measure in concussed athletes: a prospective cohort.	No	Fair		
Pilot Randomized Controlled Trial of an Exercise Program Requiring Minimal In-person Visits for Youth With Persistent Sport-Related Concussion.	Yes		Some Concerns	
Early psychological symptoms predict concussion recovery time in middle and high school athletes.	No	Fair		

Incidence of concussion and recovery of neurocognitive dysfunction on ImPACT assessment among youth athletes with premorbid depression or anxiety taking antidepressants.	No	Fair		
Factor Structure for the Sport Concussion Assessment Tool Symptom Scale in Adolescents After Concussion.	No	Fair		
The Impact of Knee Injury History on Health-Related Quality of Life in Adolescent Athletes.	No	Poor		
Concussion Symptoms Predictive of Adolescent Sport-Related Concussion Injury	No	Poor		

Multidisciplinary Concussion Management: A Model for Outpatient Concussion Management in the Acute and Post-Acute Settings.	No		High	
Coping with sports injuries: an examination of the adolescent athlete.	No	Good		
The Association between Length of Recovery Following Sport-Related Concussion and Generic and Specific Health-Related Quality of Life in Adolescent Athletes: A Prospective, Longitudinal Study	No	Good		

The relationship between post-injury measures of cognition, balance, symptom reports and health-related quality-of-life in adolescent athletes with concussion.	No	Fair		
Predicting Wellness after Pediatric Concussion	No	Fair		
Exploring Heterogeneity of Stepped Collaborative Care Treatment Response Trajectories after Adolescent Sports Injury Concussion.	No		High	
Psychosocial correlates of young athletes' self-reported concussion symptoms during the course of recovery.	No	Fair		

Concussion Incidence, Duration, and Return to School and Sport in 5- to 14-Year-Old American Football Athletes.	No	Fair		
Association of impulsivity, physical development, and mental health to perceptual-motor control after concussion in adolescents	No	Poor		
The role of psychosocial risk factors for injury in elite youth ice hockey	No	Good		
Acute Effects of Concussion in Adolescent Athletes With High Preseason Anxiety.	No	Fair		
Evaluating Multiple Domains of Health in High School Athletes With Sport-Related Concussion.	No	Fair		

Mental health issues in elite rugby league players in the NRL during a competitive season-a cross sectional epidemiological study	No	Fair		
Factors related to the presence of head injury in bicycle-related pediatric trauma patients	No	Poor		
Investigation of the effects of sports injuries on the quality of life of adolescent athletes	No	Poor		
Subjective well-being and training load predict in-season injury and illness in youth soccer players	No	Fair		

Health-Related Outcomes 3-15 Years Following Ankle Sprain Injury in Youth Sport: What Does the Future Hold?	No	Fair		
Vulnerability and stressors on the pathway to depression in a global cohort of young athletics (track and field) athletes.	No	Fair		
Subconcussive head impact exposure, drill intensity, and mental health outcomes in high school football.	No	Fair		
A preliminary exploration of the application of self-compassion within the context of sport injury.	No	Fair		

Predictors and prevalence of postconcussion depression symptoms in collegiate athletes.	No	Fair		
Psychological predictors of injury occurrence: A prospective investigation of professional Swedish soccer players.	No	Fair		
Psychological predictors of sport injuries among junior soccer players.	No	Fair		
Psychosocial Factors and Ballet Injuries.	No	Fair		
Longitudinal Clinical and Neuroimaging Evaluation of Symptomatic Concussion in 10-to 14-year-old Youth Athletes	No	Good		

The role of family and personal psychiatric history in postconcussion syndrome following sport-related concussion: A story of compounding risk	No	Good		
Prevalence of Posttraumatic Stress Disorder Symptoms Among Young Athletes After Anterior Cruciate Ligament Rupture	No	Fair		
Thinner Cortex in Collegiate Football Players With, but not Without, a Self-Reported History of Concussion	No	Fair		
Correlation of aggressiveness and anxiety in fighting sports.	No	Poor		

A functional magnetic resonance imaging study of working memory in youth after sports-related concussion: Is it still working?	No	Fair		
Longitudinal Assessment of Depressive Symptoms After Sport-Related Concussion in a Cohort of High School Athletes	No	Good		
The Relationships Between Sport Specialization, Sleep, and Quality of Life in Female Youth Volleyball Athletes	No	Good		
Clinical predictors of vestibulo-ocular dysfunction in pediatric sports-related concussion	No	Fair		

Relationship between Standalone Performance Validity Test Failure and Emotionality among Youth/student Athletes Experiencing Prolonged Recovery following Sports-related Concussion	No	Fair		
High School Athletes' Health-Related Quality of Life Across Recovery After Sport-Related Concussion or Acute Ankle Injury: A Report From the Athletic Training Practice-Based Research Network	No	Fair		
Association between depressive symptoms and subsequent injuries in early adolescents: a population-based study.	No	Good		

Sports- and Physical Activity-Related Concussions, Binge Drinking and Marijuana Use among Adolescents: The Mediating Role of Depression and Suicidal Ideation.	No	Fair		
Head Impact Exposure in Youth Soccer and Variation by Age and Sex	No	Fair		
Sports- or Physical Activity "Related Concussions and Feelings of Sadness or Hopelessness Among U.S. High School Students: Results From the 2017 Youth Behavior Risk Survey.	No	Fair		

A season long investigation into the effects of injury, match selection and training load on mental wellbeing in professional under 23 soccer players: A team case study	No	Fair		
Psychological Consequences of Athletic Injury among High-Level Competitors	No	Fair		
An examination of psychosocial variables moderating the relationship between life stress and injury time-loss among athletes of a high standard	No	Poor		
Subjective well-being is associated with injury risk in adolescent elite athletes	No	Fair		

Supplementary Material 5. Funnel plot of studies included in the first meta-analysis.

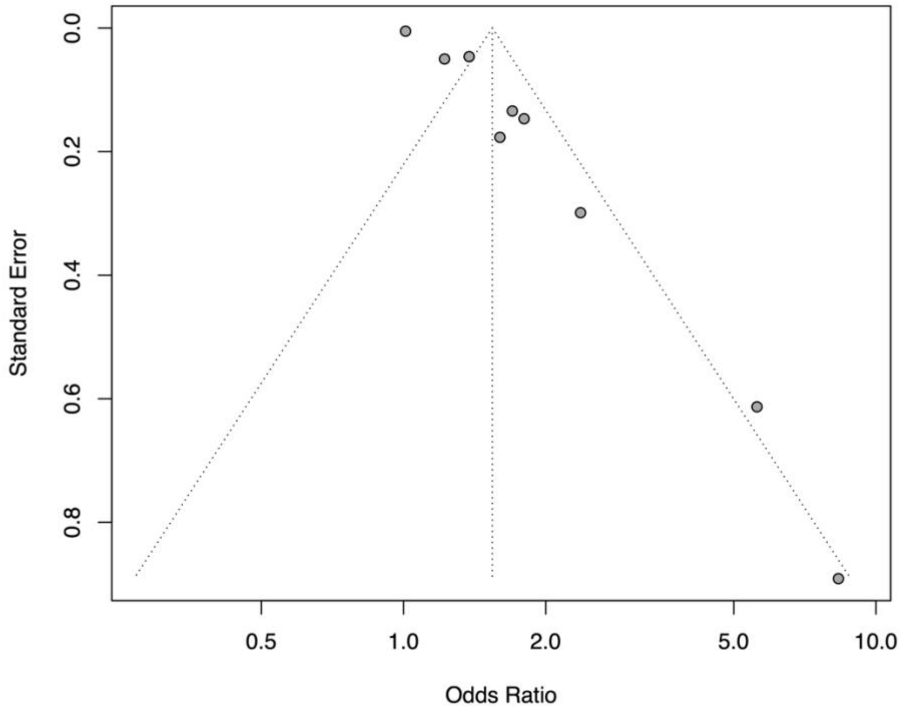

Supplementary Material 6. Funnel plot from the first meta-analysis, following trim and fill procedure.

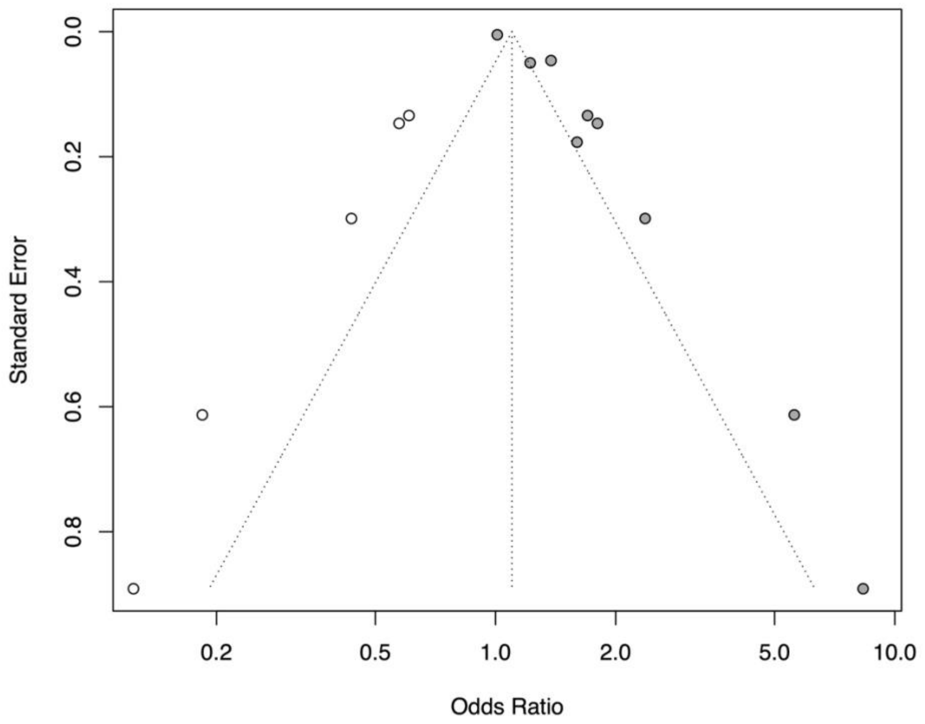

Supplementary Material 7. Funnel plot of studies included in the second meta-analysis.

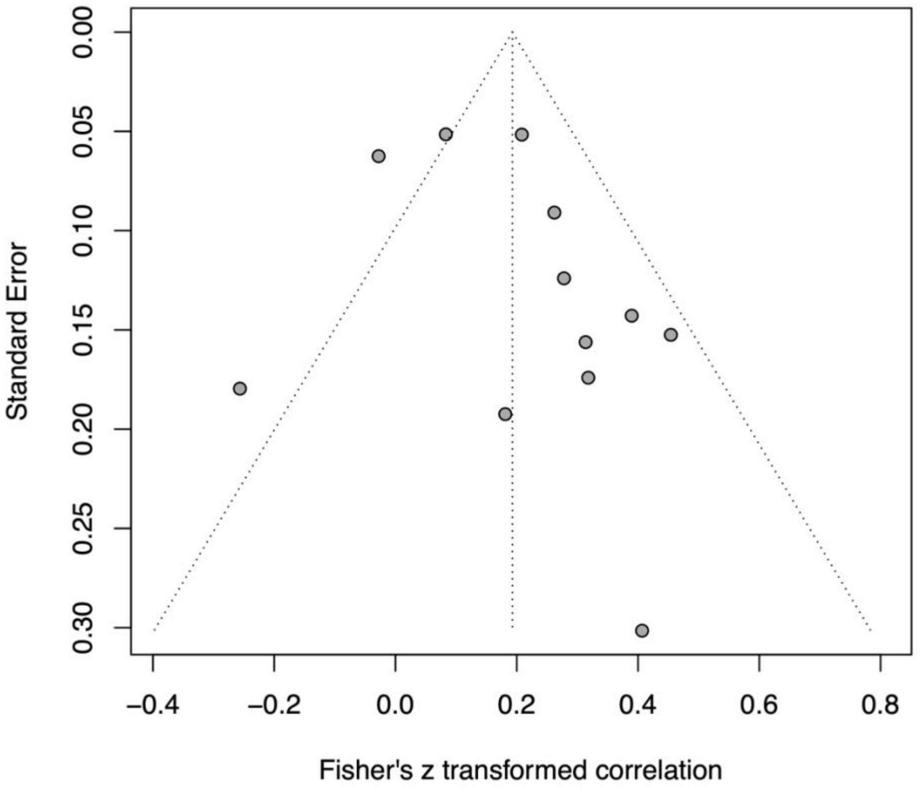

Supplementary Material 8. Funnel plot from the second meta-analysis, following trim and fill procedure.

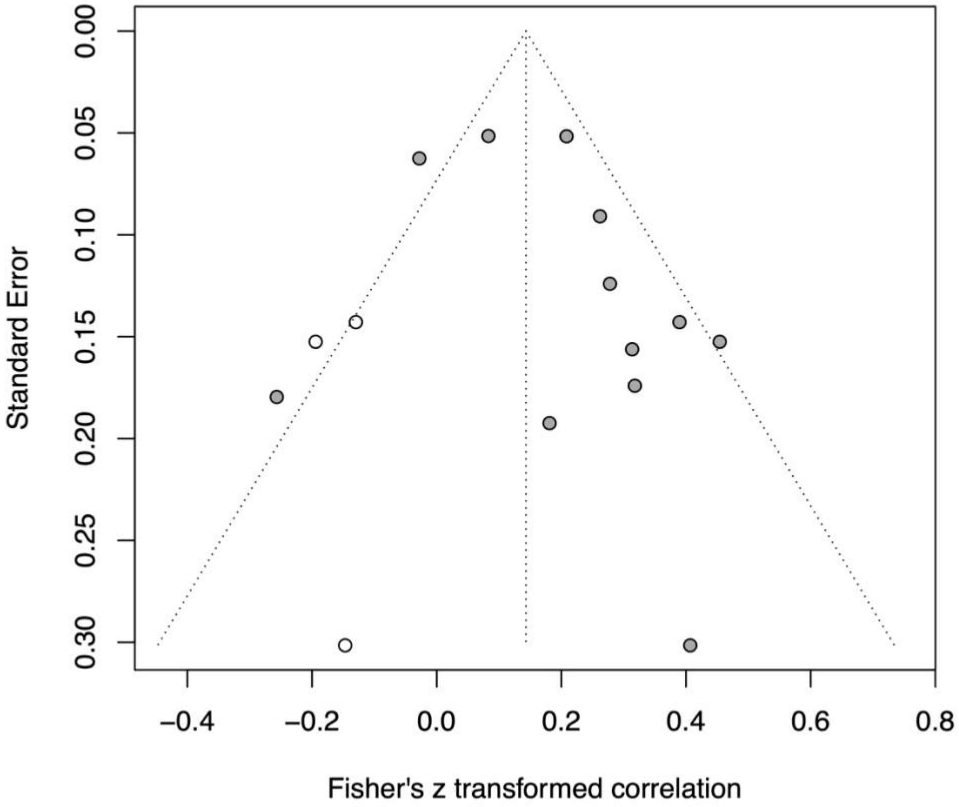

Supplementary Material 9. Funnel plot of studies included in the third meta-analysis.

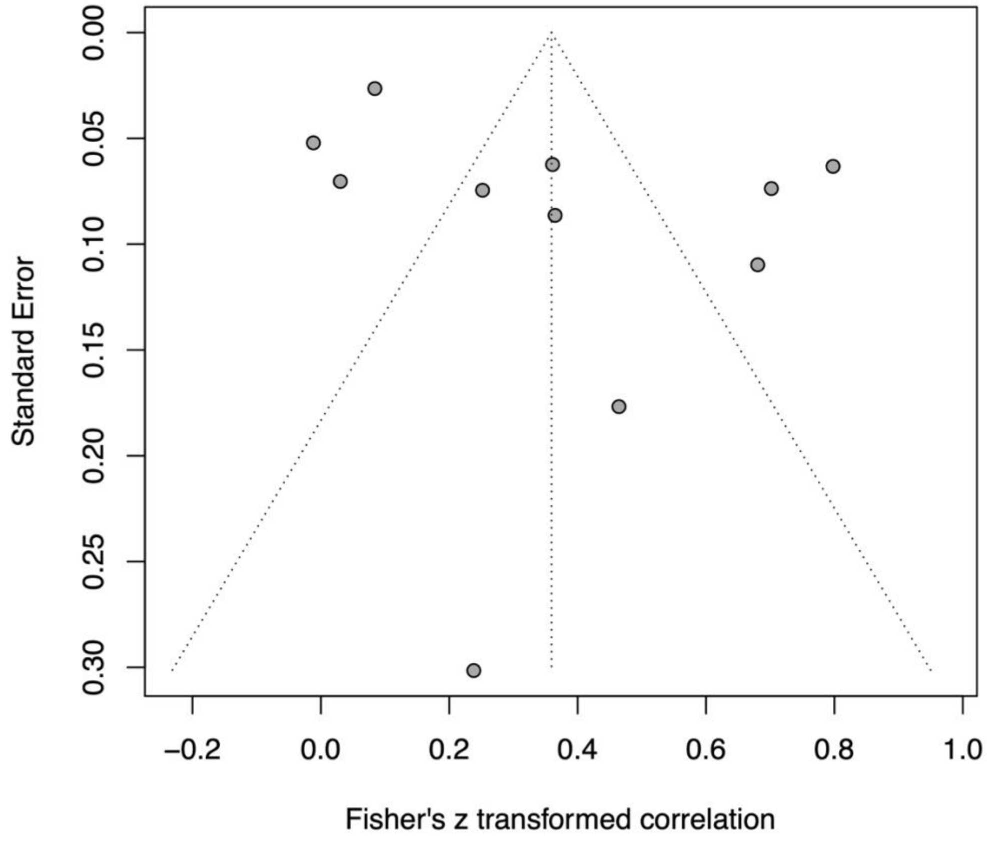

Supplementary Material 10. Funnel plot from the third meta-analysis, following trim and fill procedure.

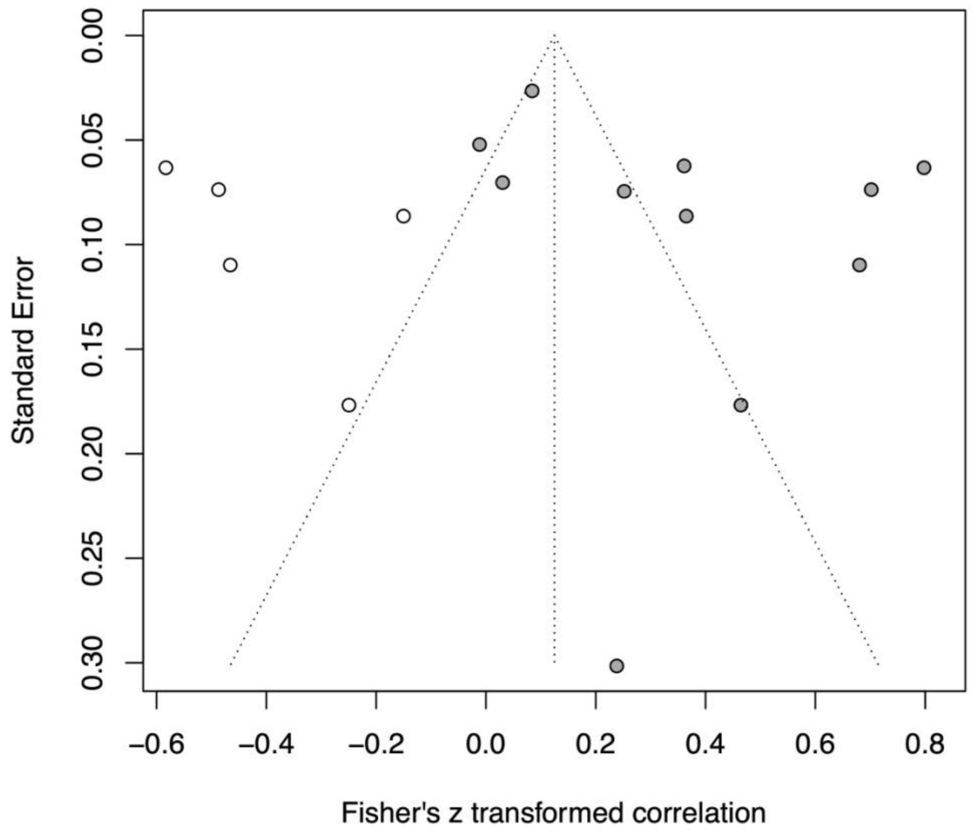

Supplement: Supplementary file 1 — Supplementary file1 (PDF 864 KB) [file 40279_2025_2379_MOESM1_ESM.pdf]
